# Effect of Al-Doping of Faceted SrTiO_3_ on Surface Chemistry in Photocatalytic Oxidation of Acetone

**DOI:** 10.1021/acs.jpcc.6c02976

**Published:** 2026-09-03

**Authors:** Nathália T. Costa, Afroditi Triptsi, Daniel M. Cunha, Shirsendu Das, Jorik Schaap, Joachim F. Woitok, Annemarie Huijser, Jitte Flapper, Georgios Katsoukis, Guido Mul

**Affiliations:** † Photocatalytic Synthesis Group, MESA^+^ Institute for Nanotechnology, 3230 University of Twente, P.O. Box 217, 7500 AE Enschede, The Netherlands; ‡ Inorganic Material Science Group, MESA^+^ Institute for Nanotechnology, 3230 University of Twente, P.O. Box 217, 7500 AE Enschede, The Netherlands; § Industrial Focus Group XUV Optics, MESA^+^ Institute for Nanotechnology, 3230 University of Twente, P.O. Box 217, 7500 AE Enschede, The Netherlands; ∥ Akzo Nobel Decorative Coatings B.V., P.O. Box 3, 2171 AJ Sassenheim, The Netherlands

## Abstract

We have investigated how Al^3+^ doping affects the facet-specific performance of SrTiO_3_ in gas-phase photocatalytic oxidation of acetone, comparing cubic and truncated structures. In situ Diffuse Reflectance Fourier Transform Infrared Spectroscopy (DRIFTS) indicates that Al-doping suppresses facet-specific adsorption of acetone and results in very similar relatively weakly adsorbed states of acetone on both, cubic and truncated structures. Oxidation of adsorbed acetone on both Al^3+^ doped SrTiO_3_ structures proceeds via α-cleavage, yielding acetaldehyde and formaldehyde, followed by the formation of surface-bound acetate and formate. The absence of CO_2_ suggests these species deactivate the surface leading to rapid termination of the oxidation process. Interestingly, the rate at which surface bound products are formed appears higher for the Al^3+^-doped truncated SrTiO_3_ than for the cubic morphology—contrary to the undoped analogues showing higher rates for the cubic structure. Time-resolved Photoluminescence (TRPL) analysis shows that Al^3+^ doping enhances charge carrier lifetimes in both morphologies, compared to undoped SrTiO_3_ having the same morphologies. Importantly, the Al-doped cubic STO shows shorter carrier lifetimes (τ_1_ = 86 ± 0.1 ps; τ_2_ = 709 ± 1 ps) compared to the Al-doped truncated material (τ_1_ = 116 ± 0.1 ps; τ_2_ = 1070 ± 1 ps)—which is consistent with the observed faster acetone transformations for the latter. This study shows Al^3+^ doping of SrTiO_3_ induces a transition from crystallographically controlled, to defect-dominated surface chemistry upon illumination, prohibiting complete mineralization of acetone, despite a doping induced lengthening of charge carrier lifetimes of cubic and truncated SrTiO_3_ structures.

## Graphical abstract

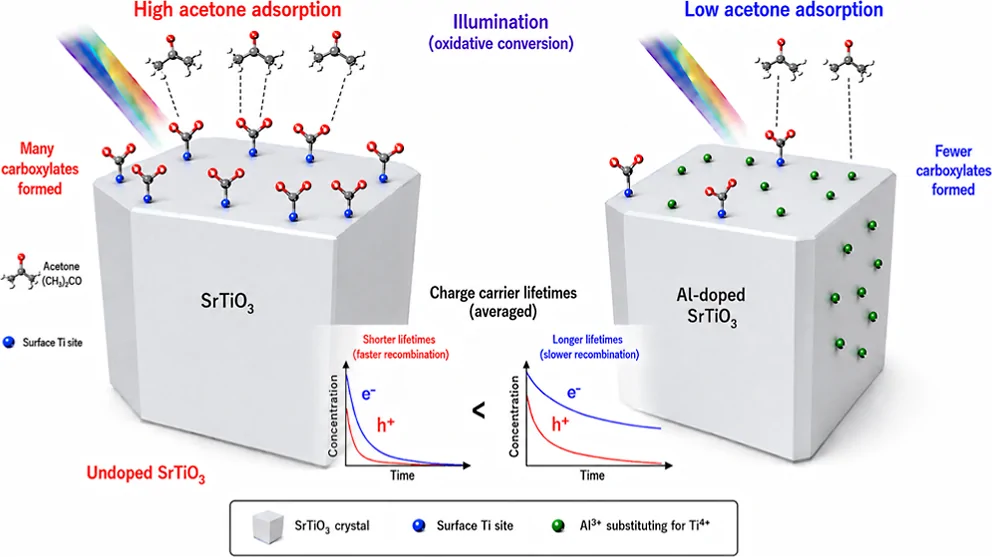

## 1 Introduction

Indoor air pollution causes significant health concerns, which necessitates the development of advanced and sustainable purification technologies. Among these, the photocatalytic oxidation of volatile organic compounds (VOCs) to CO_2_ (and H_2_O) is promising. In particular, the photocatalytic oxidation of acetone, a representative VOC, has been extensively studied, both in relation to practical relevance, as well as to gaining understanding of fundamental photocatalytic mechanisms.^1,2^

Besides TiO_2_, by far the most applied and investigated photocatalyst, strontium titanate (SrTiO_3_) is promising, particularly since (i) very high apparent quantum efficiencies have been reported in water splitting and (ii) structuring of SrTiO_3_ in the form of cubes or dodecahedra is relatively straightforward, allowing investigation of facet-specific performance in photocatalytic reactions.^3^

Structuring of SrTiO_3_ is achieved by hydrothermal or solvothermal synthesis methods, where the choice of alcohol as a solvent or cosolvent influences the final shape of SrTiO_3_ by selectively interacting with specific facets, altering their relative growth rates. Studies have shown that alcohols with lower p*K*_a_ values, such as 1,3-propanediol, stabilize high energy {110} facets, leading to truncated morphologies, while high p*K*_a_ alcohols, like ethanol, favor low-energy {100} facets, forming cubic particles.^4,5^ Having both facets exposed enhances photocatalytic performance, explained by migration of electrons to {100} facets, facilitating the reduction of adsorbed O_2_ to ^•^O_2_^–^, while holes are proposed to migrate to {110} facets, where oxidation of adsorbed water generates ^•^OH radicals.^6,7^

The different crystal morphologies of SrTiO_3_, which vary in the ratio of exposed {100} and {110}, also influence the sorption properties of SrTiO_3_. A previous study in our group identified that the {100} facets contain a higher quantity of surface hydroxyl groups compared to {110} facets, resulting in two distinct, facet-dependent modes of acetone adsorption on the photocatalyst surface.^8^ Acetone adsorption on Ti-terminated {100} facets occurs through the η^1^-enolate form, leading to the formation of μ^2^-formate and μ^2^-acetate. While μ^2^-formate is decomposed into CO_2_ and water via reactions with ^•^OH, μ^2^-acetate is photostable. In contrast, acetone adsorption on {110} facets occurs through the η^1^-acetone form, leading to production of surface formaldehyde through methyl intermediates and μ^2^-acetate.^8^

SrTiO_3_ typically exhibits oxygen vacancies accompanying the presence of Ti^3+^ sites. In photocatalytic applications, Ti^3+^-associated sites are proposed to induce electron–hole recombination, thereby lowering apparent quantum efficiencies. Doping SrTiO_3_ with aliovalent metal cations, such as Al^3+^, has been shown to enhance its photocatalytic performance significantly in the overall water splitting reaction, reaching a close to unity apparent quantum efficiency. This is explained by a reduced amount of Ti^3+^-associated oxygen vacancies, caused by Al^3+^ doping, thereby increasing the separation efficiency of photogenerated carriers.^7,9–13^

This study investigates the effect of Al doping of SrTiO_3_-faceted crystals on the surface chemistry associated with photocatalytic oxidation of acetone, via *in situ* diffuse reflectance infrared Fourier transform spectroscopy (DRIFTS). Photophysical characterization was performed by time-resolved photoluminescence (TRPL) spectroscopy to study the effect of Al^3+^ doping on lifetimes of photoexcited charge carriers. We will demonstrate that Al^3+^ doping significantly alters adsorption of acetone on the surface facets of SrTiO_3_ and results in very similar, relatively weakly adsorbed states of acetone on both cubic and truncated structures. We will also show that Al^3+^ doping enhances the charge carrier lifetimes in both morphologies investigated. We will briefly discuss how these findings are relevant to the development of efficient materials for photocatalytic air purification technologies.

## 2 Experimental Methods

### 2.1 Hydrothermal Synthesis of SrTiO_3_

The synthesis of undoped SrTiO_3_ having different proportions of {100} and {110} facets is described in detail elsewhere.^8^ The cubic-STO ({100} facet exposed only) and {110} truncated rhombic dodecahedra-STO are labeled c-STO and trd-STO, respectively, throughout this study.

### 2.2 Solid-State Synthesis of Al-Doped SrTiO_3_

The Al-doped SrTiO_3_ samples (c:Al-STO and trd:Al-STO) were prepared by solid-state synthesis as described by Ham et al.^10^ Pristine SrTiO_3_ (c-STO and trd-STO), previously synthesized using the hydrothermal method,^8^ were used as substrates. SrCl_2_ (≥99.99% trace metals basis, Sigma-Aldrich), Al_2_O_3_ (Sigma-Aldrich Co, LLC., nanopowder), and SrTiO_3_ were combined in a molar ratio of 10:0.02:1 (on a 1 g SrTiO_3_ basis) and ground thoroughly in an agate mortar. The resulting mixture was transferred to an alumina crucible and heated at 1100 °C (ramp rate 10 °C min^–1^) for 10 h in a tube furnace, flowing air at a rate of 100 mL min^–1^. After cooling naturally to room temperature, the product was washed three times with distilled water to remove residual SrCl_2_ and other soluble byproducts, continuing until the pH of the supernatant was neutral. To detect any unreacted SrCl_2_, a qualitative test using an aqueous solution of 1% AgNO_3_ was performed. To this end, after washing the photocatalyst to remove soluble residues, a few drops of the liquid were added to 5 mL of the AgNO_3_ solution. The presence of chloride ions (Cl^–^), indicative of SrCl_2_, was determined by observing the formation of a white precipitate of silver chloride (AgCl) due to the reaction of ${SrCl}_{2\left(aq\right)}+2{AgNO}_{3\left(aq\right)}\to 2{AgCl}_{\left(s\right)}+Sr{\left({NO}_{3}\right)}_{2\left(aq\right)}$ . Finally, the Al:SrTiO_3_ powders were dried overnight at 70 °C in an oven.

## 3 Characterization of the Materials

### 3.1 X-ray Diffraction and Rietveld Analysis

XRD was employed to assess the crystallographic structure of the samples. The XRD patterns of all synthesized materials were recorded in the 2θ range from 20 to 80° using a Bruker D2 PHASER X-ray powder diffractometer with Cu Kα (λ = 1.5418 Å) X-ray radiation, under an acceleration voltage of 30 kV.

The XRD data were analyzed by means of Pawley fits.^14^ A Pawley fit extracts the peak intensity from an indexed powder diffractogram. This method offers almost all possibilities of a Rietveld refinement but does not require crystal structure data. It requires only cell parameters of all present phases and their space group settings. The XRD peak positions are calculated and refined from the unit cell and known symmetry. The peak width is refined by the Caglioti^15^ function, and the shape is described by a polynomial function changing slowly with 2θ. Each single-peak intensity is refined independently. The analysis was performed with the software HighScore.^16^

#### 3.1.1 Scanning Electron Microscopy/Energy-Dispersive Spectroscopy

The morphology of the synthesized materials was analyzed by using SEM. SEM images were recorded at the MESA+ NanoLab facility using a Zeiss Merlin HR-SEM, equipped with an EDS accessory for elemental composition analysis. The EDS system, provided by Oxford Instruments, featured Vantage software and an X-Max 80 detector. Measurements were performed using an electron beam of 10 kV and a current of approximately 200 pA with area scans employed to analyze the regions of interest.

### 3.2 Raman Spectroscopy

Raman spectroscopy measurements were performed with a Senterra Bruker instrument, equipped with a cooled charge-coupled device detector. The spectra were recorded at λ_ex_ = 532 nm, 3 s integration time, 5 coadditions, 10 mW laser power, and 3–5 cm^–1^ resolution. The quantification of species was performed on baseline-corrected spectra normalized to the intensity of the respective most intense peak.

### 3.3 In Situ Diffuse Reflectance Infrared Fourier Transform Spectroscopy (DRIFTS)

*In situ* DRIFTS data were obtained by a Bruker Vertex 70 spectrometer equipped with a liquid N_2_-cooled mercury cadmium telluride (MCT) detector and a Harrick Praying Mantis diffuse reflectance accessory containing a three-window cell, two of which consist of CaF_2_, and one of quartz (through which illumination of the sample is performed). The full procedure of acetone adsorption and acetone photocatalytic oxidation has been described in detail elsewhere.^8^

### 3.4 Photophysics of Al-Doped SrTiO_3_ with Different Crystal Morphologies

#### 3.4.1 Time-Resolved Photoluminescence (TRPL) Experiments

The experimental setup used for photoluminescence experiments has been described in detail elsewhere.^8^ Briefly, the output of a Fianium laser (FP-532-1-s, 532 nm center wavelength, 300 fs pulse duration, and 80.37 MHz repetition rate) was used to activate (“pump”) the sample. For experiments with λ_exc_ = 267 nm, a second harmonic UV signal was generated by focusing 700 mW into a 3 mm thick β-BaB_2_O_4_ crystal (Newlight Photonics) using a 20 cm focal length quartz length and recollimated after the second harmonic generation. The λ_exc_ = 267 nm experiments were performed using a power of 27 μW. The PL signals were collected and focused on the input of a spectrograph (Acton SP2300, Princeton Instruments, 50 lines/mm grating blazed at 600 nm) with two 2 in. focal glass lenses (50 mm focal length). Prior to the time-resolved PL experiments, the spectral calibration was checked and adapted if necessary, using an Hg/Ar calibration lamp (Oriel, LSP035). To account for the spectral sensitivity of the setup, a spectral sensitivity correction was performed based on the measured and provided emission spectrum of a blackbody calibration lamp (Ocean Optics, HL-2000).

The open-source program Pyglotaran^17,18^ was used to perform global analysis, using a sequential model. The spectrotemporal PL behavior could be described with two pathways for all of the studied materials. To determine the final lifetime values, multiple iterations were performed until the point at which the values stabilized (i.e., changed less than the error).

## 4 Results and Discussion

### 4.1 Characterization of the Materials

#### 4.1.1 X-ray Diffraction and Rietveld Analysis

The crystal structure and phase composition of all synthesized materials were determined through X-ray diffraction analysis. The XRD patterns of the undoped SrTiO_3_ (c-STO and trd-STO) and the Al-doped SrTiO_3_ (c:Al-STO and trd:Al-STO) are shown in Figure 1. The sharpness of the diffraction lines indicates the presence of a well-ordered crystalline material. All the diffraction patterns match the standard pattern of SrTiO_3_ (JCPDS No. 35-0734) and are in agreement with the diffraction patterns reported by Ham et al.^10^ Additionally, significant shifts to higher 2θ angles are observed for the c-STO and c:Al-STO, potentially attributed to changes in lattice parameters or slight instrumental variations during measurements. Pawley refinement of the measured XRD patterns was performed to determine the exact lattice parameters and the various bond lengths in the crystal structure.

**Figure 1:**
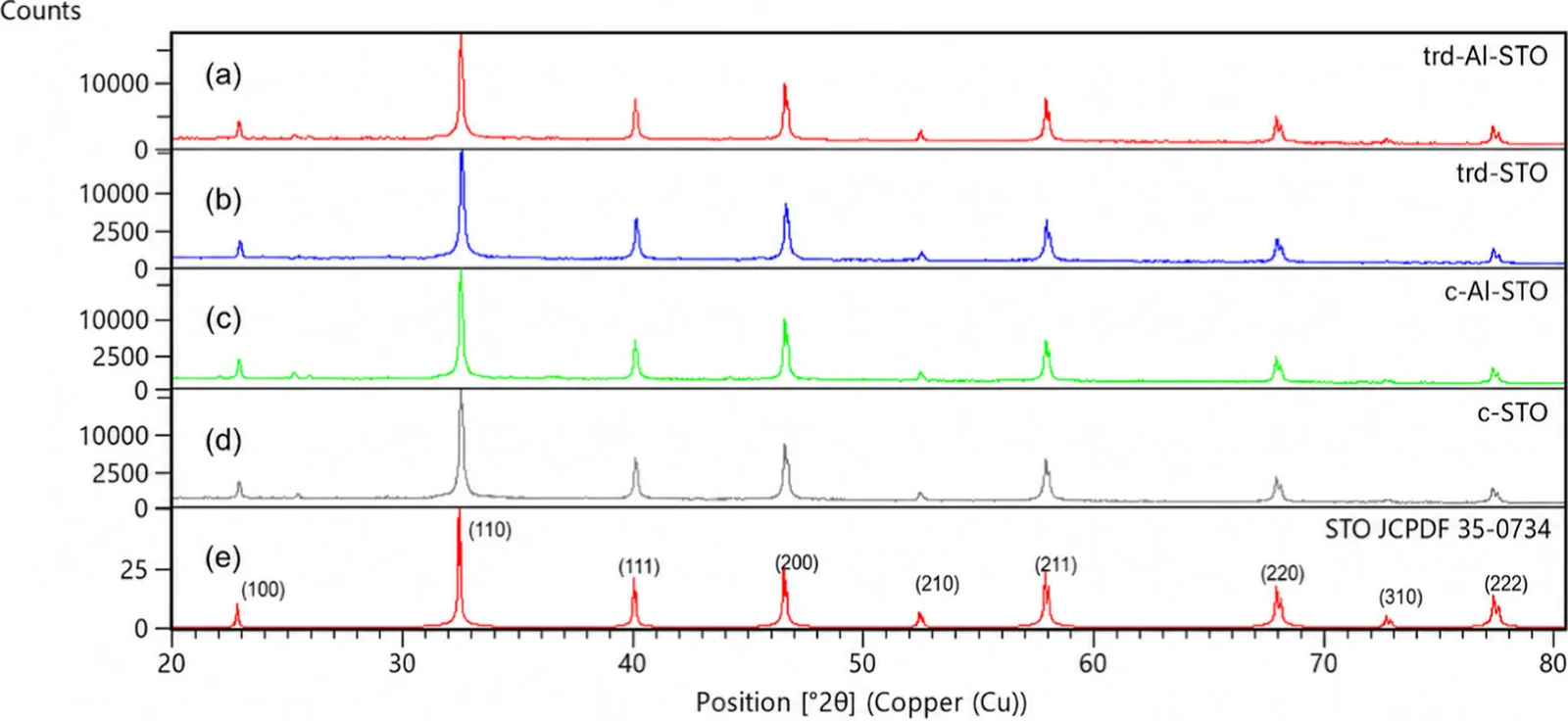
XRD patterns of (a) trd:Al-STO, (b) trd-STO, (c) c:Al-STO, (d) c-STO, and (e) standard cubic SrTiO_3_ (JCPDS No. 35-0734), with index planes labeled.

The refinement for c-STO is depicted in Figure 2 as an example. All remaining refinements can be found in the Supporting Information (Figure S1).

**Figure 2:**
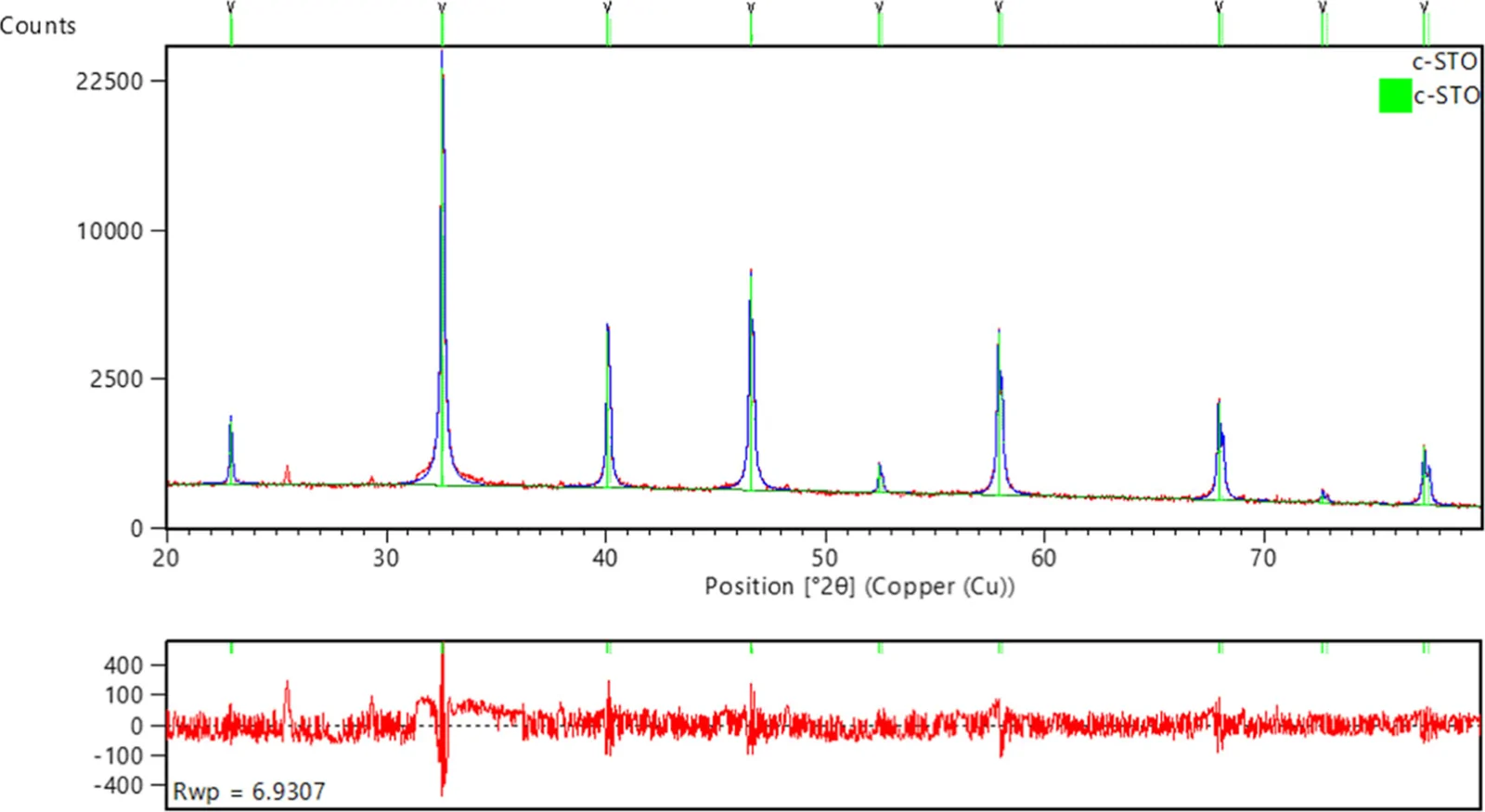
Refined Pawley fit of the XRD pattern of c-STO with the corresponding residual plot (bottom).

Table 1 summarizes the refined values of lattice parameters, fwhm of the (200) Bragg line, and the corresponding goodness-of-fit indicator (*R*_wp_ value) for each sample. A low *R*_wp_ value corresponds to better agreement between the observed and calculated diffraction patterns, indicating a more-accurate structural model.

**Table 1:** Refined Values of Lattice Parameters, fwhm of (200) Bragg Peak and the Corresponding *R*_wp_ Value for Each Sample

description	c-STO	c:Al-STO	trd-STO	trd:Al-STO
lattice parameter (Å)	3.90626	3.90552	3.90575	3.90543
Std Dev.	0.00003	0.00003	0.00004	0.00003
fwhm (200) (β rad × 10^–3^)	1.84	1.64	1.83	1.43
weighted profile *R*-value *R*_wp_	6.9307	8.8466	7.1108	8.1777

We observe that trd-STO has a smaller lattice parameter than does c-STO. The results also reveal that Al doping reduces the lattice parameter for c-STO as well as trd-STO, which is confirmed by the refined fwhm values of the (200) Bragg line.

#### 4.1.2 Scanning Electron Microscopy/Energy Dispersive Spectroscopy

Figure 3a shows an image of the cubic Al-doped sample (c:Al-STO), while Figure 3b shows a representative image of Al-doped truncated rhombic dodecahedra particles (trd:Al-STO). Figure S2a,b show the pristine c-STO and trd-STO, respectively, used for synthesis of the Al-doped samples. The small rounded particles observed in the image of both pristine surfaces (shown in the SI in Figure S2a,b) are TiO_2_ impurities.^9^ These TiO_2_ impurities are only present in minor quantities (if any) in the Al-doped STO samples. The removal of TiO_2_ impurities may be attributed to the high-temperature calcination at 1100 °C in the presence of SrCl_2_, which likely induces solid-state conversion of residual TiO_2_ facilitating the phase-pure formation of SrTiO_3_, without loss of crystal facet morphology. Previous studies have shown that flux-assisted synthesis, particularly with chloride salts, significantly improves crystallinity and phase purity by lowering reaction barriers and stabilizing the desired perovskite structure during high-temperature treatment.^19,20^ Importantly, Al-incorporation did not significantly alter the morphology, as both doped samples retained their characteristic shape of their pristine SrTiO_3_ precursors.

**Figure 3:**
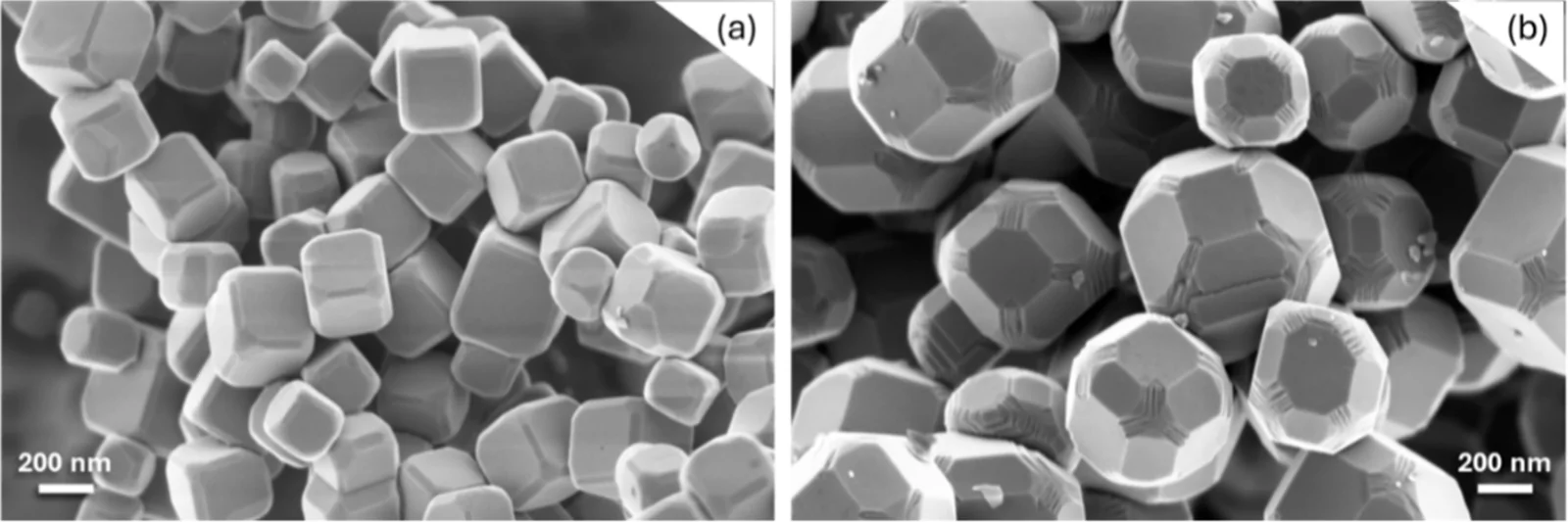
SEM images of SrTiO_3_ particles (a) c:Al-STO and (b) trd:Al-STO. The SEM images of the precursor samples are provided in the Supporting Information (Figure S2).

The EDS analysis presented in Figure S3 confirmed aluminum incorporation in both doped samples, with concentrations ranging between 0.4 and 0.7%. Additionally, minor amounts of carbon were detected, likely due to the adhesive tape used during sample preparation for SEM analysis.

#### 4.1.3 Raman Spectroscopy

Raman spectroscopy was employed to investigate the structural modifications induced by Al doping in cubic and truncated SrTiO_3_ photocatalysts. Figure 4 shows the comparison between the respective samples. Vibrational mode assignments are compiled in Table 2.

**Figure 4:**
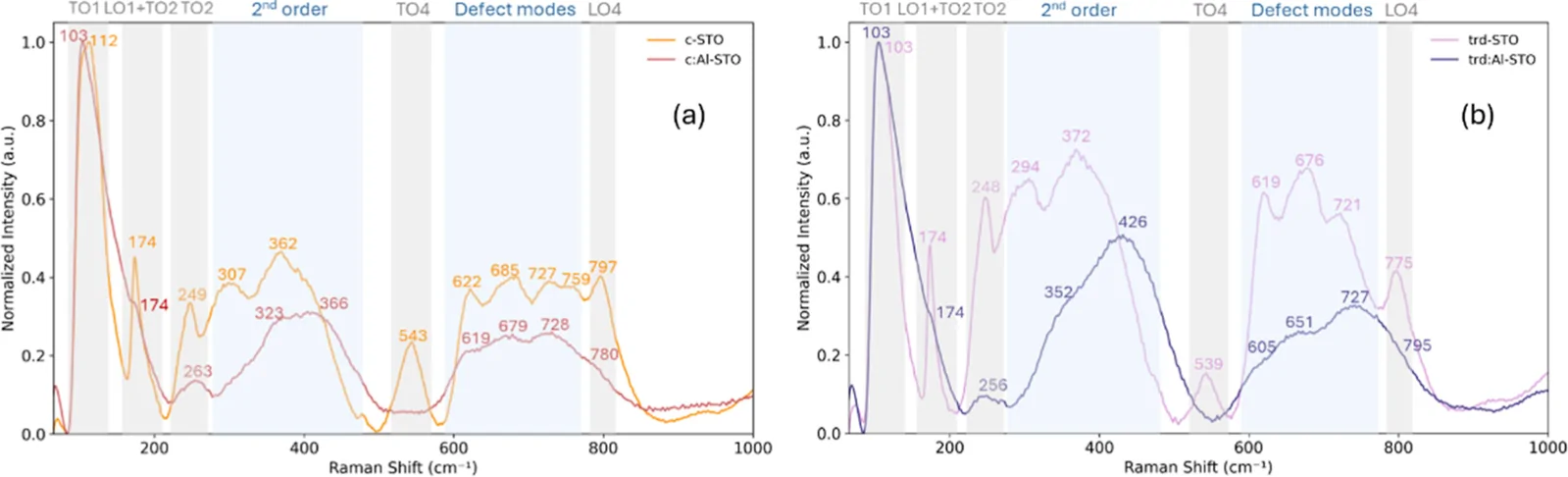
Raman data comparing the undoped and doped morphologies; (a) c-STO and c:Al-STO and (b) trd-STO and trd:Al-STO.

**Table 2:** Raman Peak Assignments According to Refs 21–28

mode (cm^–1^)	c-STO (cm^–1^)	c:Al-STO (cm^–1^)	trd-STO (cm^–1^)	trd:Al-STO (cm^–1^)	description
TO1 (∼100–120)	112	103	114	114	Ti–O bond bending/soft mode (ferroelectricity)
LO1 + TO2 (∼170)	174	174	174	174	Ti–O lattice vibrations, disorder-related
TO2 (∼250–260)	249	263	248	256	octahedral tilting/distortions
2nd order (∼300–370)	307	323	294	294	Ti–O vibrations, defects
	362	366	352	372	
TO4 (∼540)	543	-	539	-	Ti–O stretching vibrations
defect modes (∼600–800)	622	619	619	605	Ti–O stretching distortions or multiphonon
	685	679	676	651	
	727	728	721	727	
	759	780	-	775	
LO4 (∼790–800)	795	-	794	-	Ti–O longitudinal optical phonon

Figure 4 shows that both undoped samples (c-STO and trd-STO) exhibit characteristic Raman features typically forbidden for the ideal cubic phase (*Pm*3̅*m*); these are visible due to lattice distortions, oxygen vacancies, and surface defects.^21–24^ Upon Al doping, both c:Al-STO and trd:Al-STO exhibit systematic peak shifts and broadening compared to the respective undoped material, especially in the TO2 (∼250–260 cm^–1^) and second-order (∼300–370 cm^–1^) regions. These blue-shifts reflect local compressive strain in the TiO_6_ octahedra, attributed to the substitution of Ti^4+^ (0.605 Å) by the smaller Al^3+^ cations (0.535 Å).^25,26^ This observation is further supported by our XRD refinement results, which show a reduction in lattice parameters for both undoped SrTiO_3_ samples following Al incorporation as previously discussed. Similar doping-induced shifts have been reported for Fe-doped SrTiO_3_ systems, supporting the distortion mechanism.^27,28^ Murthy and coauthors^29^ also discuss that Al doping modifies the Raman spectrum of SrTiO_3_. Although these authors only show a limited region of the Raman spectrum, they attribute a shoulder at approximately 800 cm^–1^ to vibrational modes associated with the local structural environment around Al^3+^ ions. This is inconsistent with our spectral observations: the 797 or 775 cm^–1^ Raman bands weaken in intensity, rather than increase in intensity.^29^ This is likely due to the much more ordered structures (cubes) used in this study, which modify the vibrational properties of the lattice. Accordingly, the blue shifts observed in the TO2 and second-order Raman regions in our study are consistent with local structural perturbations introduced by Al doping. Furthermore, the reduction in Raman intensity and peak broadening, particularly at higher wavenumbers (600–800 cm^–1^), indicates an enhanced concentration of structural defects, likely associated with oxygen vacancy formation.^21,23,30^ The doping effects are more pronounced in c:Al-STO, for which the undoped crystal (c-STO) initially possesses higher lattice symmetries and fewer defects, making it more susceptible to distortion.

These observations confirm successful Al incorporation into both SrTiO_3_ morphologies, accompanied by comparable local structural distortions and increased oxygen vacancy concentrations, likely affecting the adsorption behavior of VOCs (acetone), as we will demonstrate next.^7,31,32^

### 4.2 In Situ Diffuse Reflectance Infrared Fourier Transform Spectroscopy

#### 4.2.1 Acetone Adsorption

Diffuse reflectance infrared Fourier transform spectroscopy (DRIFTS) was employed to investigate the consequences of Al doping on the adsorption of acetone. The comparative DRIFT spectra in the low wavenumber range between each respective undoped|doped pair are displayed in Figure 5. Please note in Figure 5b, the *y*-axis has a 3-fold lower absorbance quantity. The full wavenumber range spectra of both Al-doped STOs are shown in the Figure S5 in the Appendix.

**Figure 5:**
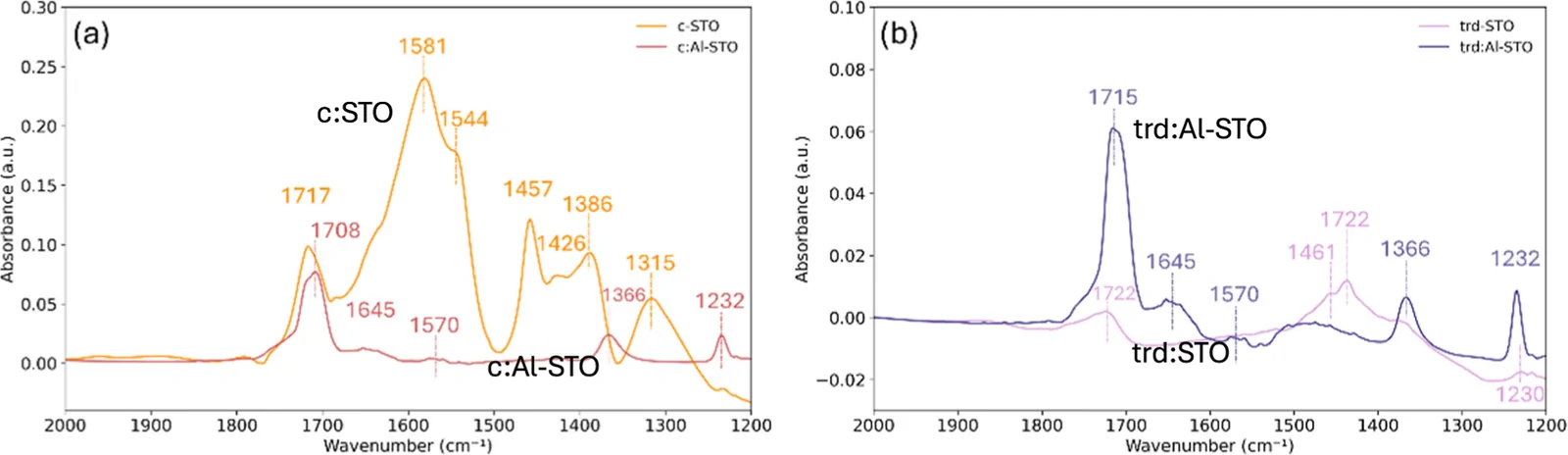
In situ DRIFT spectra of comparing acetone adsorption on the undoped and doped samples (a) c-STO and c:Al-STO and (b) trd-STO and trd:Al-STO, in the low wavenumber range (2000–1000 cm^–1^). The spectra of undoped samples are identical to those previously reported in ref 8.

Clearly high quantities of adsorbed acetone are formed on cubic STO. The main observation is that acetone adsorbs in the form of η^1^-enolate species, as evidenced by the appearance of ν­(C=C) stretching modes around 1570–1580 cm^–1^,^8^ enolate formation being associated with large quantities of surface OH groups. Al doping (Figure 5a, red trace) significantly reduces the quantity of adsorbed acetone in the form of enolate. More critically, the enolate-related C=C band (∼1580 cm^–1^) is nearly absent in the doped sample, indicating a suppression of enolate formation. This suppression likely arises from a reduction in surface hydroxyl groups which strongly contribute to acetone enolation. The methyl bending modes (δ_CH_3__) in the 1380–1450 cm^–1^ region also exhibit systematic shifts, particularly a ∼20 cm^–1^ red shift in c:Al-STO, highlighting changes in acetone adsorption geometry and coordination environment. Thus, infrared spectra of adsorbed acetone reveal that Al doping suppresses strong acetone binding for the c-STO.

In contrast to the pristine crystals, the Al-doped trd-SrTiO_3_ exhibits significant infrared absorbance for adsorbed acetone, indicating a higher overall adsorption capacity. The spectra after doping are dominated by a strong band at ∼1715 cm^–1^ corresponding to weakly bound or molecular acetone, while features associated with strongly coordinated species are comparatively less prominent. This suggests that Al doping of trd-STO increases the density of adsorption sites—likely through the formation of oxygen vacancies and surface defects. As a result, acetone adsorption is enhanced in quantity but weakened in interaction strength, favoring more physisorbed-like species over strongly chemisorbed configurations.

These findings align with literature reports where dopant-induced modifications in metal oxides influence surface acidity, basicity, and oxygen vacancy concentrations, thereby impacting adsorption behavior.^33–35^ A related study investigating La-doped ZnSnO_3_ nanosheets for acetone sensing^36^ provides valuable insights into how cation doping can modulate surface properties through defect engineering. Using a combination of experimental techniques and Density Functional Theory (DFT) calculations, the authors demonstrated that La doping substantially increases the concentration of surface oxygen vacancies. These findings emphasize the broader principle that cation doping not only introduces lattice distortions but also tailors surface reactivity via defect-mediated electronic modulation.

In summary, these DRIFTS results show that Al doping in SrTiO_3_ reduces the ability to form enolate intermediates for c-STO, accompanied by significantly lower quantities of acetone weakly interacting with the surface, while for trd-STO Al doping enhances the quantity of adsorbed acetone, interacting with the surface comparably. Concluding, Al doping suppresses facet-specific adsorption of acetone and results in very similar relatively weakly adsorbed states of acetone on both cubic and truncated structures.

#### 4.2.2 In Situ Photocatalytic Oxidation of Acetone

The strongly decreasing facet specific adsorption is also reflected in surface selectivity when performing photocatalytic oxidation, contrary to what was observed for the pristine samples. Figure 6a,b and 6c,d show *in situ* DRIFT spectra obtained during photodegradation of surface adsorbed acetone over the two Al-doped SrTiO_3_ photocatalysts in the full (4000–1000 cm^–1^) and low (1800–1200 cm^–1^) wavenumber ranges. A compilation of observed frequencies for photodegradation of acetone on the surface of each material are shown in Table S1 in the SI.

**Figure 6:**
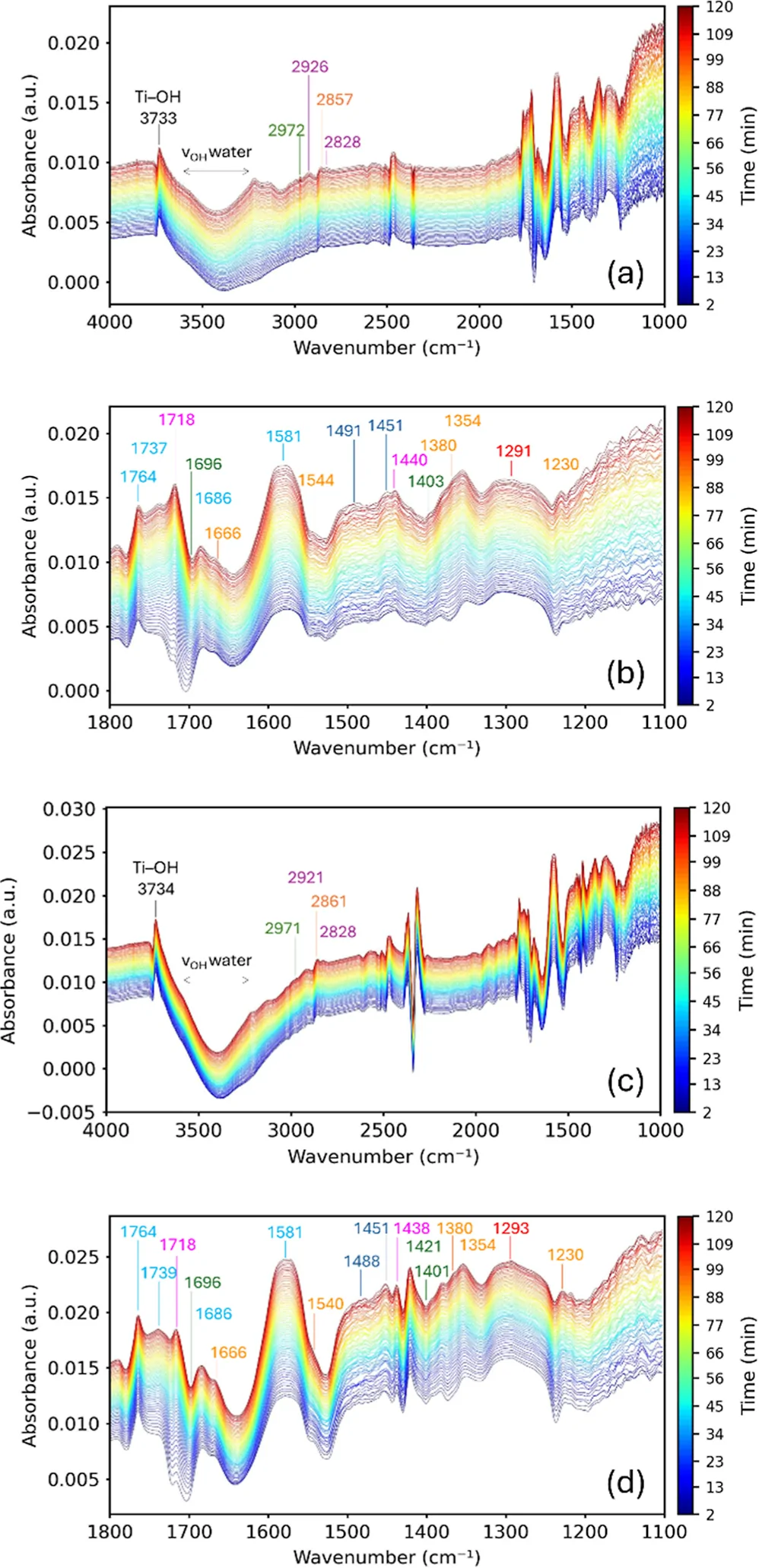
In situ DRIFT spectra of photooxidation of acetone in the full and low spectral range on (a, b) c:Al-STO; (c, d) trd:Al-STO, respectively. A high-power light-emitting diode UV light chip diode (370 nm, 1.5 mW/cm^2^) was employed for the light exposition time from 2 to 120 min.

The spectra can be explained by photocatalytic oxidation of weakly surface-bound acetone (η^1^-acetone) detectable by decreasing C=O stretching vibrations at 1696 cm^–1^; resulting in vibrational modes at 1718, 1739, and 1764 cm^–1^, assigned to aldehydes, and a broad feature centered at the 1581 cm^–1^ region, typically assigned to carboxylates such as formate (HCOO^–^) or acetate (CH_3_COO^–^). Positive features in the 1450 and 1350 regions can be assigned to carbonate and bicarbonate, respectively.^37–43^ Negative features in these spectral regions are mainly methoxy (–OCH_3_) and hydroxylated surface species also play roles as transient intermediates, particularly in humid conditions,^44,45^ but are not dominant after Al-doping.

Despite the mechanistic similarity, trd:Al-STO displays enhanced reactivity compared to c:Al-STO. The temporal evolution of the DRIFT spectra reveals earlier onset and greater intensity of all key surface species on the truncated sample, particularly those attributed to acetaldehyde and formaldehyde, as evidenced by the rate of band growth. Moreover, in Figure 6c, the OH stretching region (3750–3000 cm^–1^) shows stronger and more persistent signals for trd:Al-STO when compared to the same region in Figure 6a for c:Al-STO, in stark contrast to what was observed for the nondoped analogues. The more intense acetate and formate features observed over trd:Al-STO also point to increased surface intermediate coverage, which may result from facet-specific differences in adsorption affinity or charge separation. Also, the presence of the CO_2_ band further confirms that some intermediates were fully oxidized but to much less extent when compared to its undoped sample (trd-STO) as we observed in our previous work.^8^

Collectively, these results indicate that Al doping in both cubic and truncated SrTiO_3_ favors partial oxidation pathways while reducing or even suppressing complete mineralization to CO_2_, depending on the morphology. Notably, the trd:Al-STO surface facilitates faster activation of acetone and stabilization of oxygenated intermediates such as acetaldehyde, formaldehyde, and formate, likely due to its higher exposure of {110} facets and associated defect structures. This contrasts with the pristine materials, where the crystal morphology led to distinctly different spectral features. The observed suppression of morphology-driven behavior upon doping highlights a significant interplay between the crystal structure and dopant effects in dictating VOC photooxidation surface selectivity.

Based on these findings, the photocatalytic oxidation of acetone over Al-doped SrTiO_3_ follows a complex surface-mediated mechanism involving multiple fragmentation pathways and the stabilization of partially oxidized intermediates. As illustrated in Figure 7, we propose a stepwise mechanism including acetaldehyde as a key intermediate. A possible pathway includes the following steps; acetone adsorbs onto the Al:SrTiO_3_ surface via coordination of its carbonyl group (C=O) to Ti^4+^/Al^3+^ centers. Upon UV illumination, photochemical homolytic cleavage of the C_1_–C_2_ bond occurs, yielding methyl (^•^CH_3_) and acetyl (^•^COCH_3_) radicals. The acetyl radical rapidly abstracts a proton from surface hydroxyl groups, forming surface-bound acetaldehyde. This intermediate undergoes further oxidation by photogenerated holes (h^+^) and reactive oxygen species (ROS) through two primary pathways: (i) C_2_–C_3_ bond cleavage, producing formaldehyde and methyl radical and (ii) oxidation to a surface-bridged acetate species, known for its photostability.^8,46,47^ Subsequently, formaldehyde is oxidized to formate, which is stabilized on the Al-doped SrTiO_3_ surface. We propose that the incorporation of Al^3+^ inhibits charge transfer from the STO to surface formate, thereby suppressing its further oxidation and preventing complete mineralization to CO_2_ and H_2_O, as we previously observed in undoped SrTiO_3_ samples. Alternatively, the presence of surface Al^3+^ sites could strongly enhance the bonding strength of the adsorbed formate, decreasing the rate of photoinduced consecutive oxidation to CO_2_.

**Figure 7:**
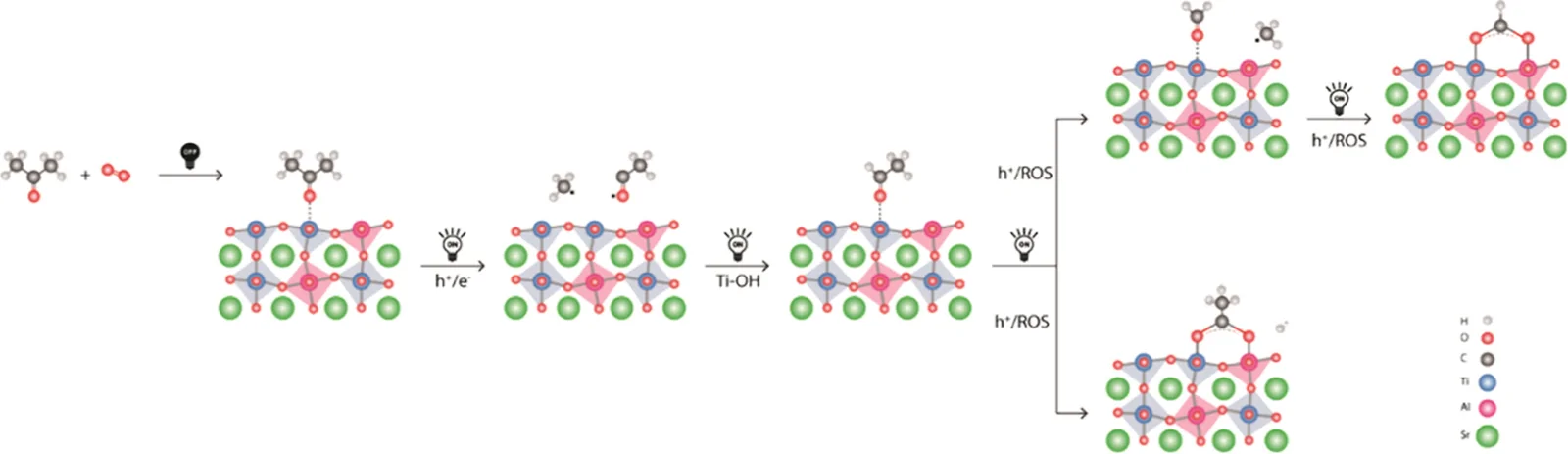
Suggested mechanism for gas-phase photooxidation of acetone on Al-doped SrTiO_3_. The Al doping modifies the oxygen orientation originating from tilting of TiO_6_ octahedra as represented here in pink. h^+^/e^–^ are holes and electrons, respectively; Ti–OH are surface OH groups; and ROS. The formation of acetate involves conversion of a superoxide anion (not shown).

Our time-resolved DRIFT spectra revealed distinct bands near ∼1764 and ∼1737 cm^–1^, consistent with adsorbed acetaldehyde, and at 1718 cm^–1^, attributed to formaldehyde. These observations strongly support our proposed reaction pathway involving initial α-cleavage of acetone followed by stepwise oxidation of acetaldehyde. The subsequent emergence of acetate and formate bands, without any detectable CO_2_ in the c:Al-STO spectra and with only trace amounts in the trd:Al-STO, indicates that oxidation proceeds selectively and likely stops at stable, surface-bound intermediates.

Furthermore, the presence of formate at early reaction stages and its high spectral intensity suggests an additional contribution from C_2_–C_3_ bond cleavage of acetaldehyde, providing a complementary route for its formation. These findings align with our previous results and further validate the proposed mechanism. The pronounced accumulation of these intermediates, on Al-doped SrTiO_3_, highlights the critical role of both surface structure and Al-induced modifications in steering the reaction mechanism away from total mineralization. Altogether, these insights emphasize how catalyst morphology and doping synergistically control oxidation selectivity and intermediate dynamics in gas-phase VOC degradation using oxide perovskite photocatalysts.

It is well known that Al doping enhances photocatalytic water splitting, where oxygen vacancies and improved charge separation play pivotal roles. The altered band structure in Al-doped SrTiO_3_ aligns better with the redox potentials for H_2_ and O_2_ evolution, while the reduced recombination rates ensure more efficient photogenerated charge-carrier utilization.^7,29^

While Al doping promotes charge dynamics favorable for water splitting, it detrimentally affects the full photooxidation of acetone by disrupting critical surface-mediated intermediate pathways. This duality provides a foundation for the rational catalyst design in distinct photocatalytic applications. To further demonstrate that Al doping promotes charge dynamics also for the samples synthesized and utilized here, we performed a time-resolved photophysical analysis.

### 4.3 Photophysics of Al-Doped SrTiO_3_ with Different Crystal Morphologies

#### 4.3.1 Time-Resolved Photoluminescence Experiments

Time-resolved photoluminescence (TRPL) measurements have been performed to investigate the photophysics of the well-faceted, Al-doped SrTiO_3_ samples. The TRPL data have been globally fitted by a two-component sequential model using Pyglotaran.^17,18^ The time-dependent spectra are shown in Figure S6, while the time-dependent traces at 420, 450, and 470 nm are shown and discussed in Figure S7. To account for the initial rise in PL, a Gaussian instrument response function (IRF) was applied. The obtained results are compared to the pristine, faceted, SrTiO_3_ we also studied in our previous work.^8^ For the discussion of the results, we focus on the traces at 450 nm of the Al-doped SrTiO_3_ morphologies and pristine analogues.

Figure 8 presents the normalized time-resolved photoluminescence (TRPL) decay traces at 450 nm for undoped and Al-doped SrTiO_3_ morphologies, obtained using 267 nm excitation. The obtained PL lifetimes are summarized in Table 3. Both Al-doped samples exhibit longer lifetimes in both morphologies, indicating suppressed electron–hole recombination upon doping. The prolonged carrier lifetime can explain the enhanced photocatalytic activity of Al-doped SrTiO_3_ in water splitting, consistent with previous reports in the literature.^6,7,10,29,48^

**Figure 8:**
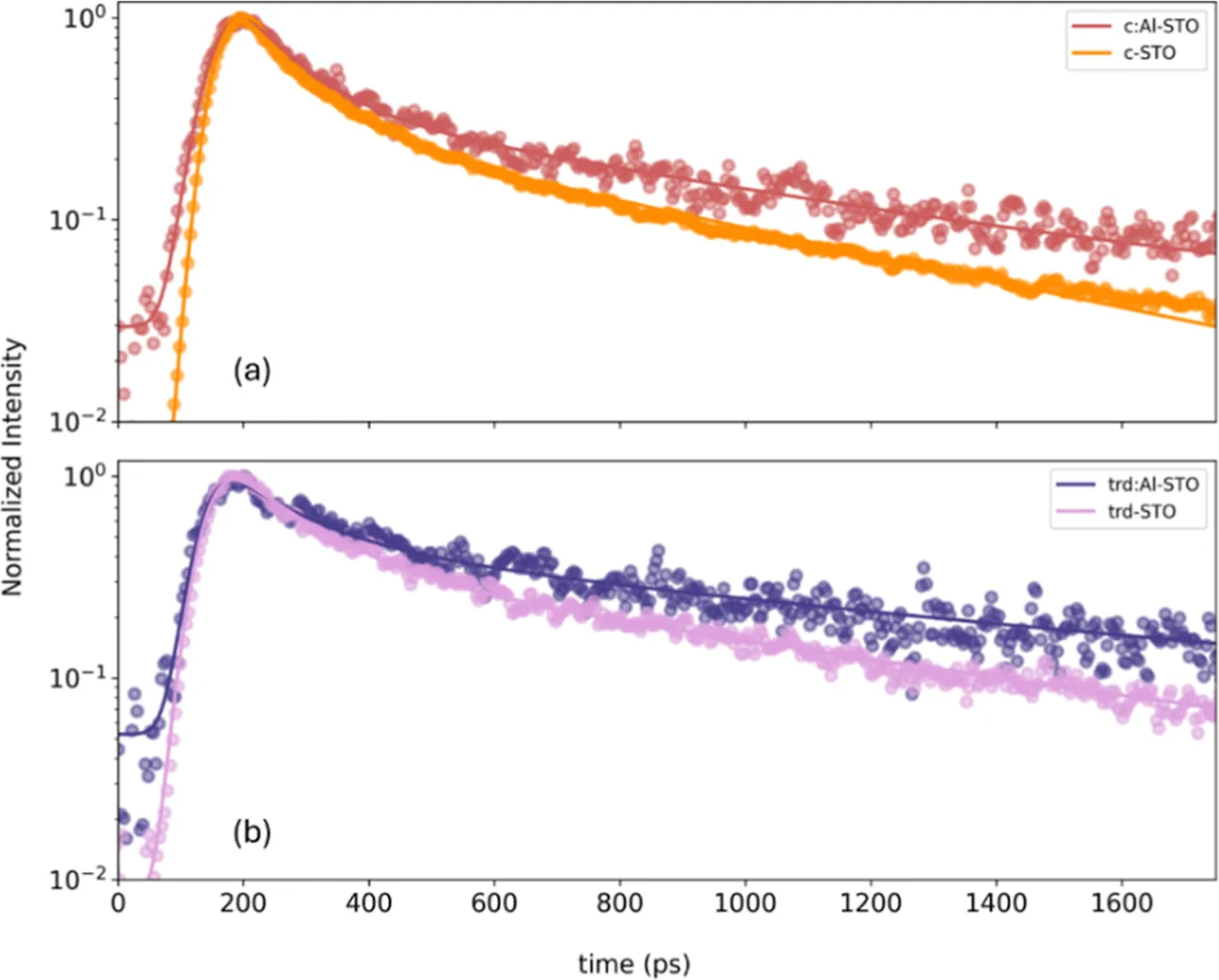
Normalized PL decays at 450 nm including fits (solid lines) for STO films following 267 nm excitation of (a) c-STO and c:Al-STO and (b) trd-STO and trd:Al-STO.

**Table 3:** Photoluminescence Lifetimes from Global Analysis using a Sequential Model with Two Components

morphology	sample	τ_1_ (ps)	τ_2_ (ps)
cubic	c-STO	81.8 ± 0.1	629 ± 1
	c:Al-STO	85.8 ± 0.1	701 ± 1
truncated	trd-STO	111.5 ± 0.1	918 ± 1
	trd:Al-STO	117.3 ± 0.1	1061 ± 1

Figure 9 shows the normalized evolution-associated spectra (EAS) corresponding to the two components obtained from our global analysis. These spectra represent the PL associated with the first (τ_1_) and second (τ_2_) recombination processes. A pronounced difference is observed between both Al-doped and the undoped SrTiO_3_ materials. In Figure 9a,b,d, the SrTiO_3_ samples exhibit only a minimal (∼5–10 nm) or nearly absent PL red shift. In contrast, for c-STO (Figure 9c), a notable red shift occurs from about 430 nm to ca. 470 nm. This spectral evolution is further detailed in Figure S6, where time-resolved PL spectra for c-STO clearly show a red shift in the PL maximum in time, from approximately 430 nm to ca. 470 nm.

**Figure 9:**
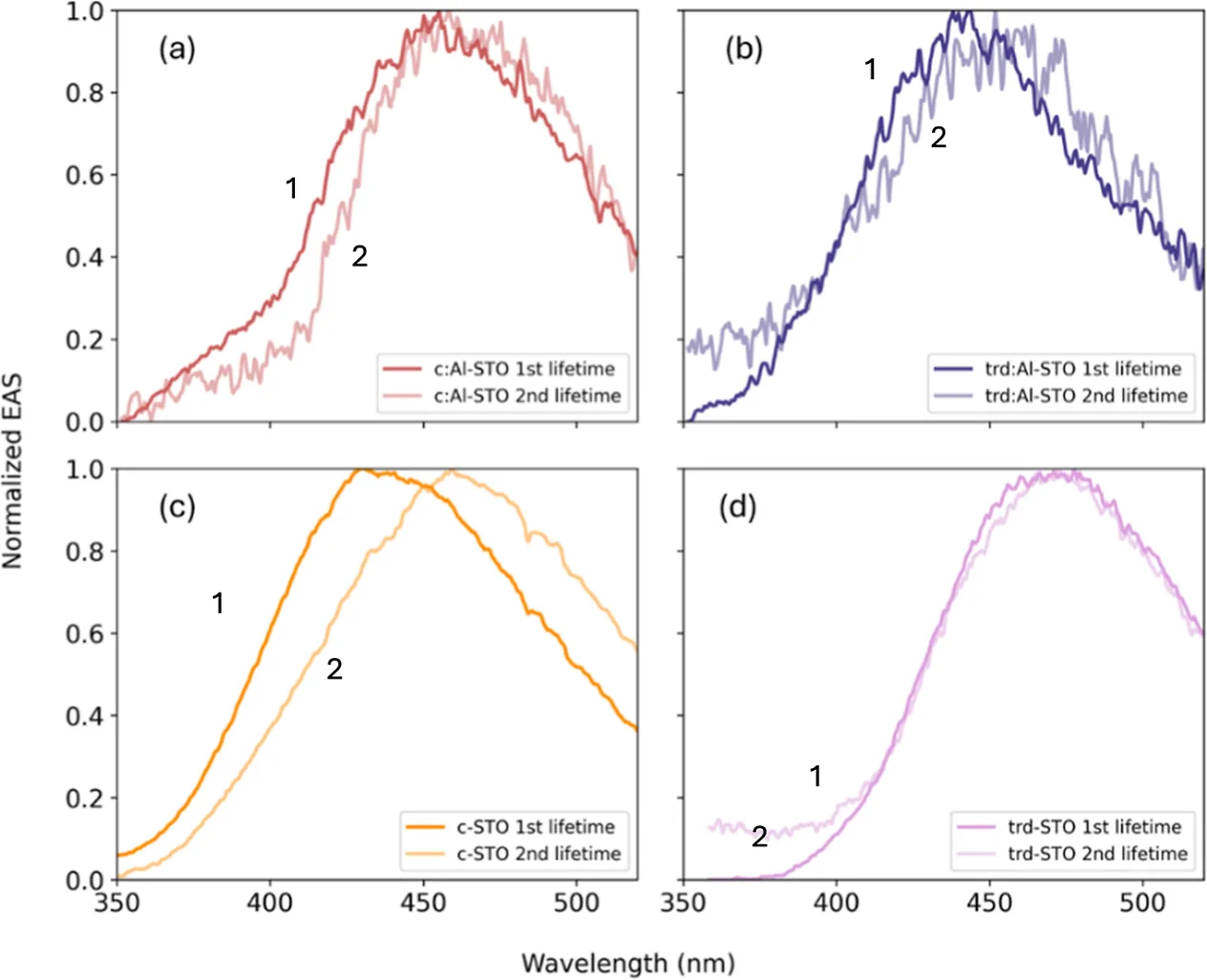
Evolution associated spectra (EAS) from global analysis using a sequential model with two components, for the four different samples investigated: (a) cubic Al:SrTiO_3_, (b) truncated Al:SrTiO_3_, (c) cubic SrTiO_3_, and (d) truncated SrTiO_3_.

The red shift in PL with time suggests electron trapping into trap states below the conduction band. A similar red shift in PL with time has also been observed for other metal oxide semiconductors like TiO_2_ and attributed to surface trap-limited recombination.^49^ The prominent red-shifted PL of ∼40 nm for c-STO suggests recombination at later times likely occurs between electrons in deep trap states with holes, whereas the small red-shift of <∼5–10 nm for trd-STO and both Al-doped STO morphologies indicates trapping of electrons in shallow trap states residing near the lower lying subconduction bands. For SrTiO_3_, these deep trap states have been reported to be Ti^3+^ defects that likely act as recombination centers.^7^ Aluminum doping of SrTiO_3_ could mitigate this surface recombination through two complementary mechanisms. First, surface trap passivation: Al^3+^ incorporation likely introduces shallow trap states that decrease the density of oxygen vacancy-related traps, effectively reducing defect-mediated recombination pathways. Second, carrier lifetime extension: Al^3+^ substitution likely increases the electron density and promotes the formation of a depletion layer near the surface, which enhances charge separation.^50,51^ These effects are consistent with the minimal PL red-shift with time observed for Al-doped SrTiO_3_, indicating suppressed surface recombination, while for trd-STO the small red-shift could be associated with shallow trap states when saturated with oxygen.

Based on these observations, we propose the photophysical model shown in Figure 10 to explain the differences in photodynamics between the undoped and Al-doped samples. Note that this model is likely a simplification of the reality, but it describes our TRPL data well. The first component in the model corresponds to PL from electron–hole recombination in the bulk shortly after photoexcitation. At this initial stage, charge carriers have not yet migrated significantly. This behavior aligns with charge carrier dynamics studies of SrTiO_3_, which indicate that a substantial fraction of photogenerated carriers recombine in the bulk at early times following excitation.^52^ The second component likely corresponds to surface recombination. Following charge separation, electrons and holes migrate toward the STO surface, potentially to different facets,^7,29^ where they can become trapped in surface states and eventually recombine.^52,53^

**Figure 10:**
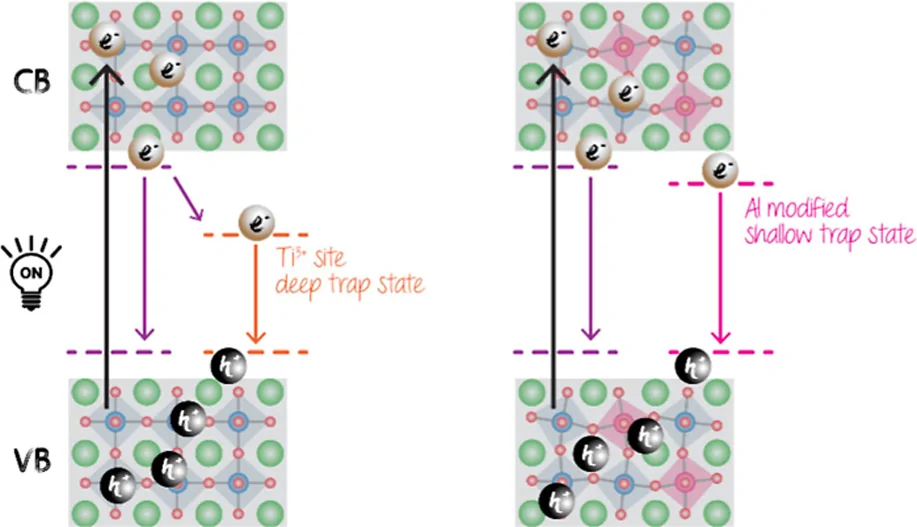
Photophysical model of the charge carrier dynamics in undoped STO (left) and Al-doped STO (right). Note that undoped STO likely has shallow rather than deep surface trap states because of saturation with oxygen. Electrons and holes are represented as e^–^ and h^+^, respectively.

Cuk et al. observed the formation of surface oxyl radical (Ti–O^•^) and the trapping of holes at these sites on ps time scales,^54^ highlighting the role of surface states in recombination processes. In undoped c-STO, Ti^3+^ defects related to oxygen vacancies likely create deep trap states near the surface of STO, yielding a significant decrease in PL lifetime and a strong PL red-shift. However, the introduction of Al^3+^ into the lattice likely replaces Ti^3+^ defects, thereby removing deep trap states and promoting charge separation.

## 5 Conclusion

This study demonstrates how facet engineering and Al doping influence the photocatalytic oxidation of acetone over SrTiO_3_-based materials. By combining *in situ* DRIFTS and time-resolved photoluminescence (TRPL), we have shown that Al^3+^ incorporation significantly alters the surface reactivity and charge carrier dynamics of both Al-doped cubic and truncated SrTiO_3_ structures when compared to the respective pristine samples. Our TRPL measurements revealed enhanced charge separation in truncated Al-doped SrTiO_3_, which correlates with the highest observed photocatalytic activity among the studied materials.

Mechanistically, a selective photooxidation pathway is proposed based on infrared spectroscopic observations that initiates via α-cleavage of adsorbed acetone, forming methyl and acetyl radicals, followed by stepwise oxidation through acetaldehyde and formaldehyde intermediates. The absence of a CO_2_ band in the DRIFT spectra of Al-doped cubic STO, and only trace amounts in the Al-doped truncated STO spectra, alongside the accumulation of surface-bound acetate and formate, strongly suggests that the reaction preferentially terminates at these stable intermediates under the tested conditions. Notably, the early and intense appearance of formate bands in both cases, likely facilitated by C_2_–C_3_ bond cleavage, reinforces the presence of parallel oxidation routes. These mechanistic insights expand upon previous findings by demonstrating how Al doping modifies both electronic properties and surface chemistry, inhibiting complete mineralization of acetone.

In summary, these results highlight the effect of Al^3+^ doping on photocatalytic performance of faceted SrTiO_3_, confirming longer lifetimes of photoexcited states, yet strongly decreasing, or even prohibiting the complete mineralization of acetone, photocatalytic oxidation terminating at strongly bound carboxylate and (bi)­carbonate species. Future work could explore the effect of humidity and/or cocatalysts to stimulate conversion of formate intermediates toward complete mineralization, as well as include detailed microscopic investigation of the particles after Al doping, such as HR-TEM to determine lattice fringes and surface defects, or AFM to atomically resolve facet-dependent hydration forces,^55^ and/or study acetone (or other hydrocarbon) adsorption at the atomic scale.

## Supplementary Material



## Notes

Refined Pawley fits of XRD patterns, additional SEM images and EDS results, description of the DRS analysis and Tauc plots, additional *in situ* DRIFT spectra, including a table with elaborate peak assignments, photoluminescence (PL) spectra, normalized PL decay curves, and short list of additional references (PDF)
