# Supplementary material for: Effect of Al-Doping of Faceted SrTiO3 on Surface Chemistry in Photocatalytic Oxidation of Acetone
Source: J Phys Chem C Nanomater Interfaces. 2026 Sep 3;130(37):12917–28. doi: 10.1021/acs.jpcc.6c02976 (PMC13592066; doi:10.1021/acs.jpcc.6c02976)
Supplement: Supplementary file 1 [file jp6c02976_si_001.pdf]

# Supporting Information

## The effect of Al-doping of Facetted $SrTiO_3$ on Surface Chemistry in Photocatalytic Oxidation of Acetone

Nathália T. Costa,[a] Afroditi Triptsi,[a] Daniel M. Cunha,[b] Shirsendu Das,[a] Jorik Schaap,[a] Joachim F. Woitok,[c] Annemarie Huijser, [a] Jitte Flapper, [d] Georgios Katsoukis,[a] Guido Mul*[a]

[a] PhD candidates N. T. Costa, S. Das, and J. Schaap;

BSc. A. Triptsi;

Prof. Dr. Annemarie Huijser; Prof. Dr. G. Katsoukis; Prof. Dr. G. Mul*

Photocatalytic Synthesis Group, MESA+ Institute for Nanotechnology,

University of Twente – P.O. Box 217

7500 AE Enschede, the Netherlands

E-mail: g.mul@utwente.nl

[b] Dr. D. M. Cunha

Inorganic Material Science Group, MESA+ Institute for Nanotechnology,

University of Twente – P.O. Box 217

7500 AE Enschede, the Netherlands

[c] Dr. J. F.W. Woitok

Industrial Focus Group XUV Optics, MESA+ Institute for Nanotechnology,

University of Twente – P.O. Box 217

7500 AE Enschede, the Netherlands

[d] Dr. J. Flapper

Akzo Nobel Decorative Coatings B.V.

P.O. Box 3

2171 AJ Sassenheim, the Netherlands

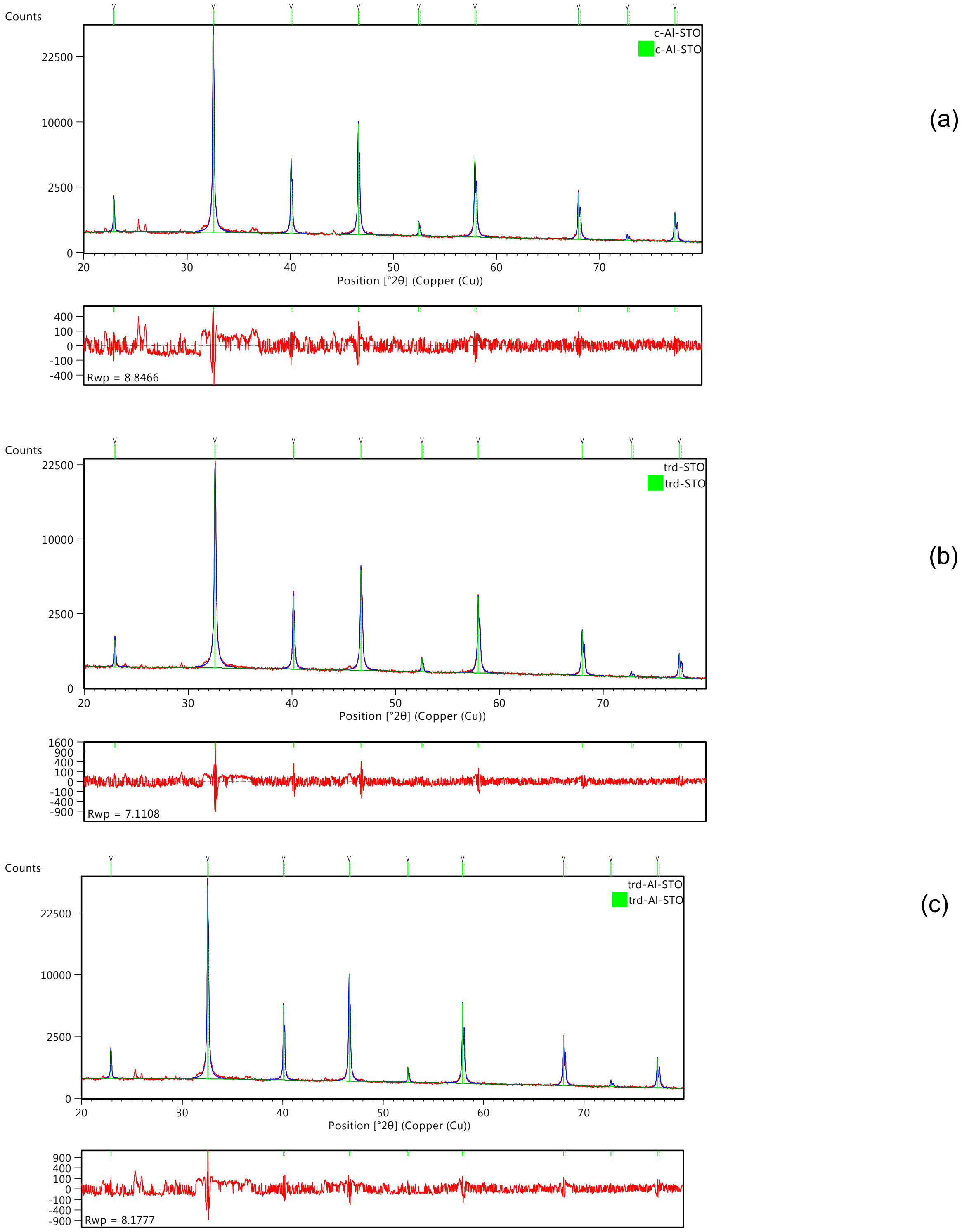


*Figure S1. Refined Pawley fit of XRD pattern of (a) c:Al-STO, (b) trd-STO and (c) trd:Al-STO with corresponding difference plot.*

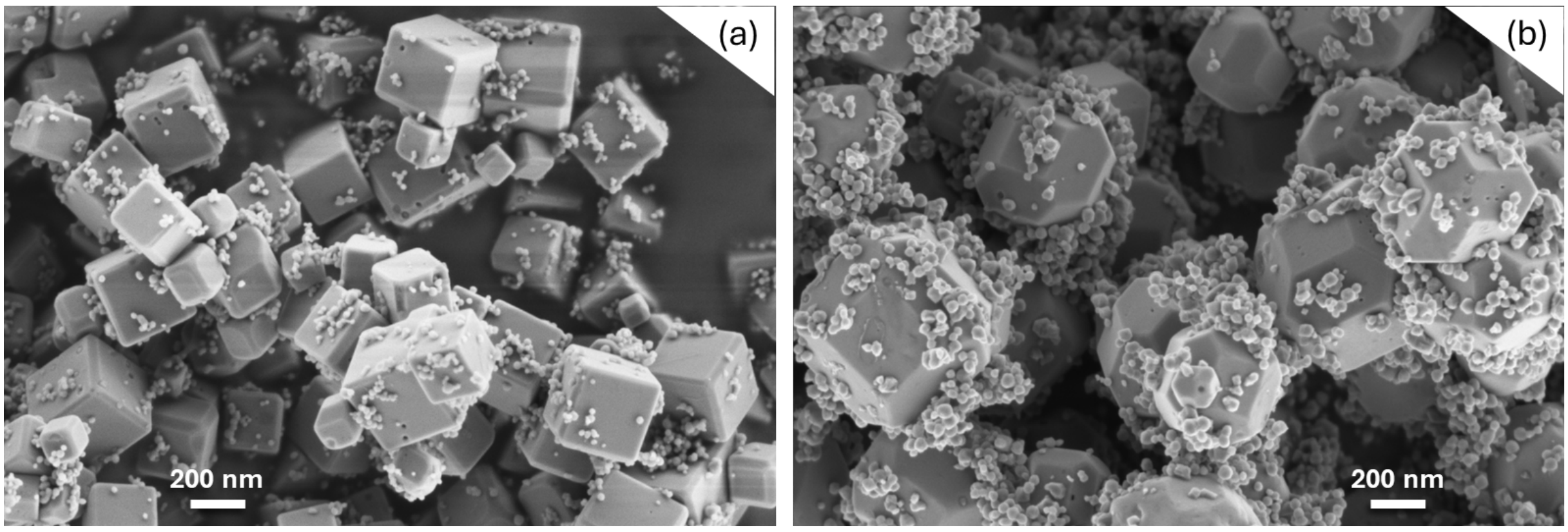


*Figure S2. SEM images of the undoped STO precursors (a) c-STO, and (b) trd-STO. The small rounded particles are $TiO_2$ impurities.*

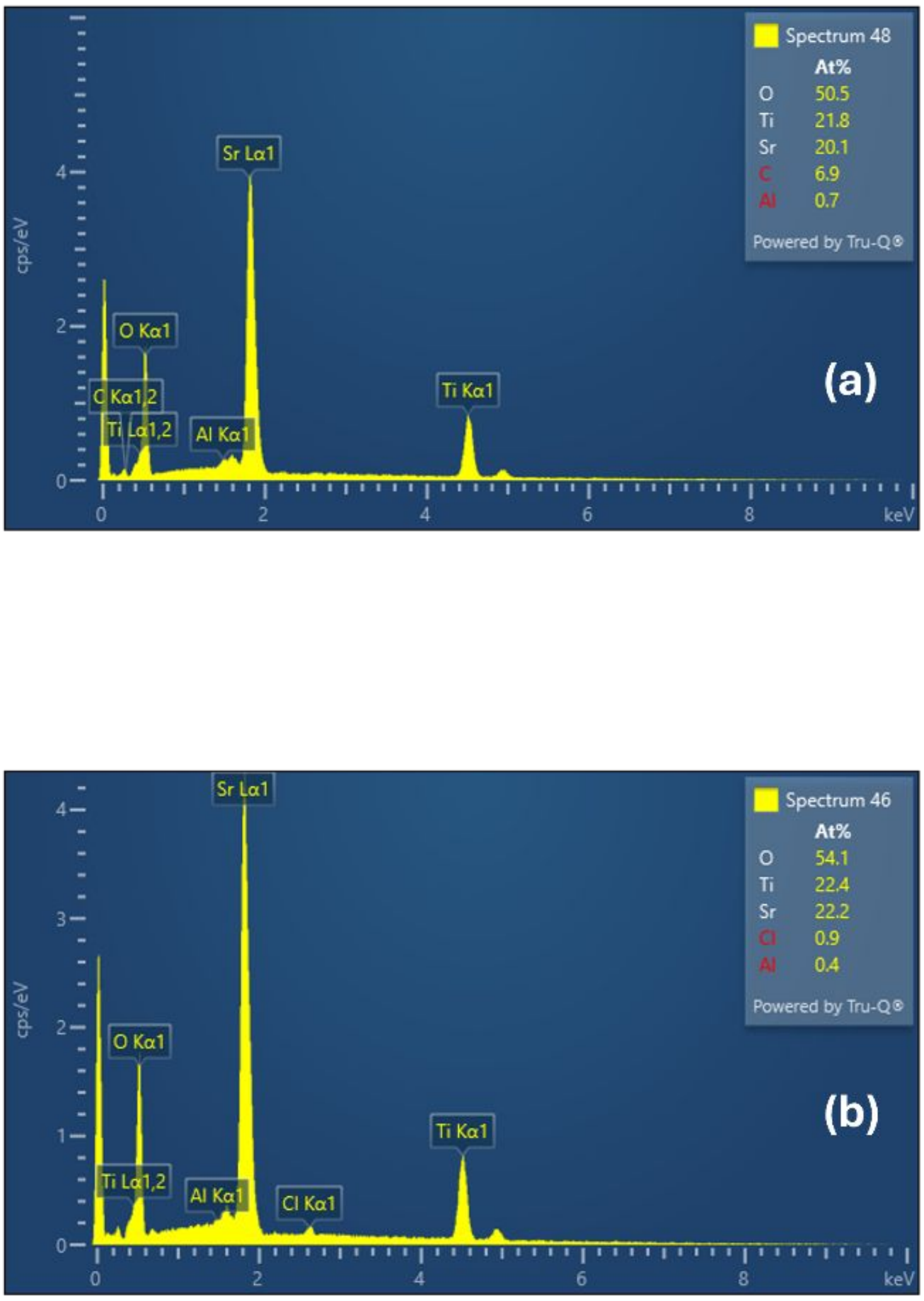


*Figure S3. EDS results of Al-doped $SrTiO_3$ particles (a) c-Al:STO, (b) trd-Al:STO showing 0.7% and 0.4% of Al content, respectively.*

### *Diffuse Reflectance Spectroscopy (DRS) & Tauc Plot*

DRS was performed using a deuterium lamp (wavelength range 215-500 nm; lamp power 78W/0.75A) of an Avantes AvaLight-DH-S-BAL light source. The determination of the reflectance spectra of the different samples was performed using a Avantes AvaSpec-2048 spectrometer. $BaSO_4$ (97%, Alfa Aesar) was used as a reference sample (100% reflectance).

The measured reflectance spectra can be transformed to the corresponding absorption spectra by applying the Kubelka−Munk function given by Equation S1:

$$F(R) = \frac{(1-R)^2}{R^2} \qquad \text{Eq. S1}$$

where R is the measured reflectance.

The Tauc plot method was employed to determine the bandgap of all materials Equation S2:

$$(a * h\nu)^{\frac{1}{\gamma}} = B\left( h\nu - E_g\right) \qquad \text{Eq. S2}$$

where α is energy-dependent absorption coefficient, h is the Planck constant, ν is the photon's frequency, E*g* is the band gap energy, and B is a constant. The γ factor depends on the nature of the electron transition and is equal to ½ or 2 for the direct and indirect transition band gaps, respectively. $SrTiO_3$ has an indirect band gap and therefore, γ was set to ½.

The Tauc plot was used to determine the band gap of all the materials. The band gap value corresponded to the point where the tangent line drawn to the point of inflection intersected with x-axis.[1]

The optical band gaps of all synthesized materials were calculated from the corresponding Tauc plots presented in Figure S3. As expected, all photocatalysts were capable of absorbing light in the region of the UV spectrum. In addition, the band gap of Al-doped STO does not significantly differ from that of the non-doped STO, suggesting that the levels of Al doping are low. This observation is consistent with the EDS results.

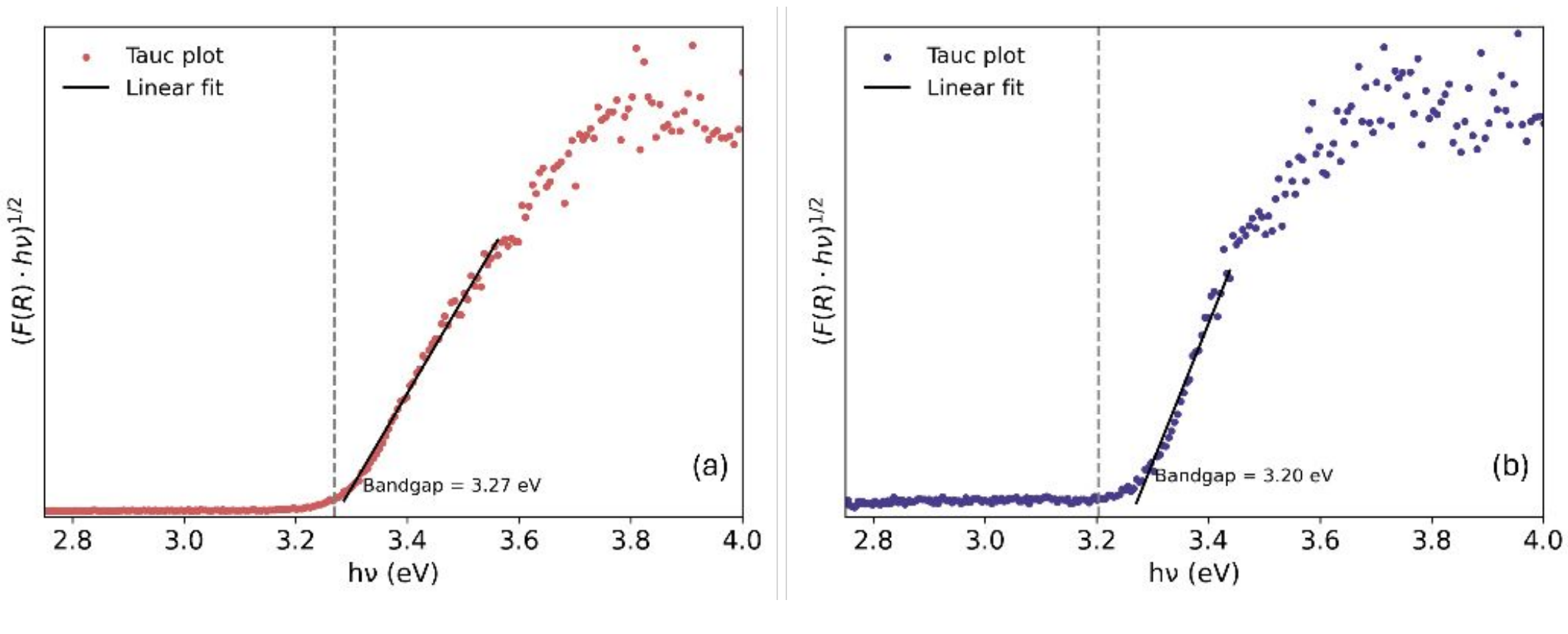


*Figure S4. Tauc plots of (a) c-Al:STO, (b) trd-Al:STO of $SrTiO_3$ particles. The linear part of the plot is extrapolated to the x-axis for band gap determination.*

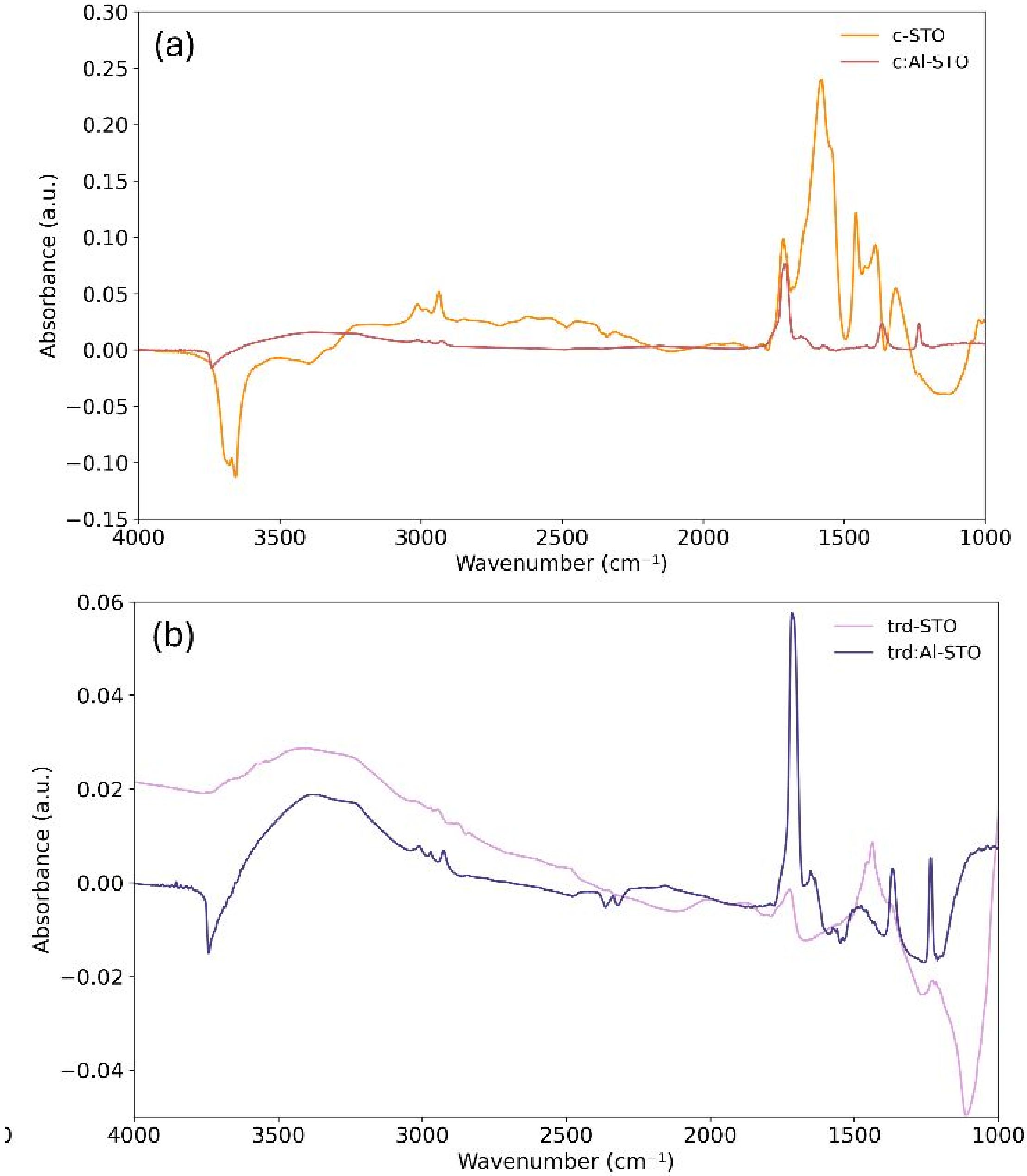


*Figure S5. In situ DRIFT spectra of comparing acetone adsorption on the undoped and doped samples (a) c-STO and c:Al-STO, (b) trd-STO and trd:Al-STO, in the full wavenumber range (4000 – 1000 cm$^{-1}$).*

*Table S1. Compilation of observed frequencies for photodegradation of acetone on c-Al:STO and trd-Al:STO, with comparison to undoped c-STO and trd-STO*

| Assignment | Type | Vibration mode | Wavenumber in this study ($cm^{-1}$) | | Wavenumber in or previous study ($cm^{-1}$) | | Wavenumber in literature ($cm^{-1}$) | Reference |
|---|---|---|---|---|---|---|---|---|
| | | | c:Al-STO | trd:Al-STO | c-STO | trd-STO | | |
| Acetone | $\eta^1$ | ν(C–H) | 2972 | 2971 | 2972 | 2960 | 3009 – 2931 | [2-5] |
| | | ν(C=O) | 1696 | 1696 | 1707 | - | 1702 – 1685 | |
| | | $\delta_{as}(CH_3)$ | 1403 | 1421 | 1420 | - | 1425 – 1419 | |
| Enolate | $\eta^1$ | ν(=C–H) | - | - | 3016 | 3016 | 3020 | [3,4,6] |
| | | ν(C=C) | 1581[a] | 1581[a] | 1581 | 1582 | 1550 | |
| | | ν(C–O) | 1291 | 1293 | 1291 | 1289 | 1300 | |
| Formate | $\mu^2$ | $\nu_s$(C–H) | 2857 | 2861 | 2873 | 2860 | 2873 | [2-5] |
| | $\mu^2$ | $\nu_s$(COO) + δ(CH) | - | | 2720 | 2773 | 2730, 2725 | |
| | $\eta^1$ | ν(C=O) | 1666[b] | 1666[b] | 1654 | 1671 | 1672 | |
| | $\mu^2$ | $\nu_{as}$(COO) | 1544[c] | 1540[c] | 1555 | 1545 | 1592 – 1542 (1578 ion) | |
| | $\eta^1$ | δ(CH) | 1380 | 1380 | 1394 | 1392 | 1381 – 1374 | |
| | $\mu^2$ | $\nu_s$(COO) | 1354 | 1354 | 1356 | 1378 | 1362 – 1363 (1414 ion) | |
| | $\eta^1$ | ν(C-O) | 1230 | 1230 | 1235 | 1234 | 1235 | |
| Acetate | $\mu^2$ | $\nu_{as}$(COO) | 1544[d] | 1540[d] | 1541 | 1535 | 1544 – 1516 | [2,7-9] |
| | $\mu^1$ | $\nu_s$(COO) | 1491 | 1488 | 1481 – 1476 | 1480 | 1504 – 1499 | |
| | $\mu^2$ | $\nu_s$(COO) | 1451 | 1451 | 1453 | 1455 | 1453 –1415 | |
| | $\mu^2$ | $\delta_s(CH_3)$ | - | - | 1334 | 1331 | 1340 | |
| Formaldehyde | | $\delta(CH_2) + \omega(CH_2)$ | - | - | - | 2703 | 2759, 2740, 2719 | [2,8-10] |
| | | ν(C=O) | 1718 | 1718 | - | 1737 | 1746, 1740, 1737, 1715 | |
| | | $\nu_s$(HCO) | 1440 | 1438 | - | - | 1413 | |
| Adsorbed methoxy | | $\nu_{as}(CH_3O)$ | 2926 | 2921 | 2925 | 2914 | 2928 | [11-13] |
| | | $\nu_s(CH_3O)$ | 2828 | 2828 | 2843 | 2845 | 2827 | |
| | | $\nu_{as}$(C-O) | - | - | 1109 | 1150 | 1115 | |
| | | $\nu_s$(C-O) | - | - | - | 1070 | 1058 | |
| Acetaldehyde | | ν(C=O); weakly adsorbed $CH_3CO$ | 1764 | 1764 | 1770 | - | 1766, 1761 | [3,14,15] |
| | | ν(C=O); H-bonded $CH_3CHO$ | 1737 | 1739 | - | - | 1736, 1733 | |
| | | ν(C=O); Ti–O=$CHCH_3$ | 1686 | 1686 | - | - | 1693 | |
| | | ν(C=O); $V_o$–O=$CHCH_3$ | 1666[e] | 1666[e] | - | - | 1677, 1675 | |
| | | $\nu_{as}$(C-O); asymmetric O–C–O stretch in coordinated $HCOO^-$ | 1581[f] | 1581[f] | - | - | 1580 | |

[a] enolate and/or [f] acetaldehyde
[b] formate and/or [e] acetaldehyde
[c] formate and/or [d] acetate

### *Time-Resolved Photoluminescence (TRPL) experiments*

In Figure S6(a), S6(b), and S6(d) of c:Al-STO, trd:Al-STO and trd-STO, respectively, exhibit only a minimal (~5-10 nm) or nearly absent red-shift. In contrast, in Figure S6(c) for c-STO, a notable red-shift occurs from about 430 nm to ca. 470 nm.

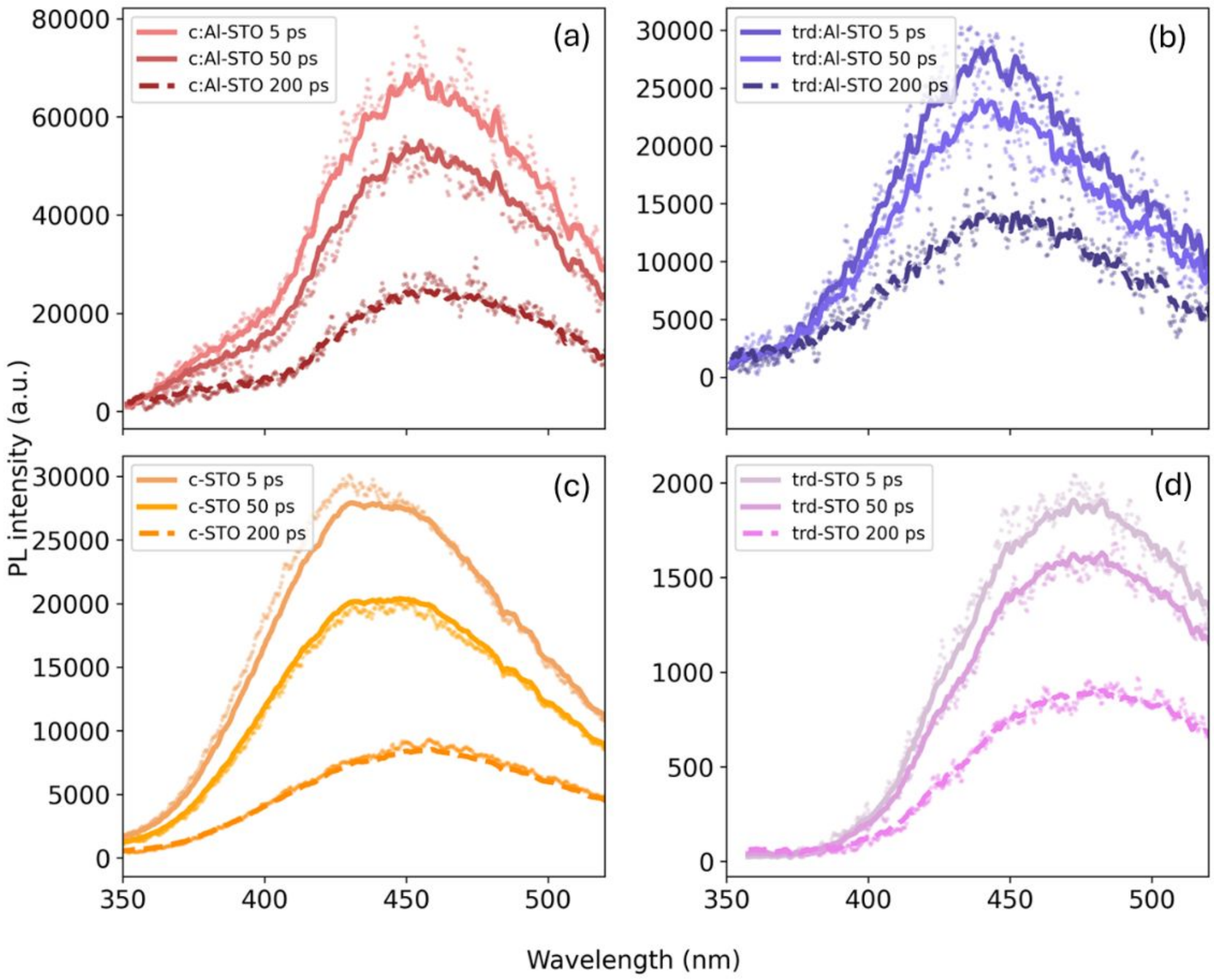


*Figure S6. Photoluminescence spectra and fits (solid and dashed lines) at different times following excitation at 267 nm at room temperature of (a) c-Al:STO, (b) trd-Al:STO, (c) c-STO, and (d) trd-STO of $SrTiO_3$ films.*

*Figure* Figure S7 compares the TRPL decay traces of Al-doped $SrTiO_3$ and undoped materials. In Figure S7(b) we observed that the trd-Al:STO exhibits a markedly longer carrier lifetime than the other STOs. As our earlier DRIFTS results indicated, the two doped morphologies share similar surface intermediates and reaction pathways, yet only the truncated form benefits significantly from Al-doping in terms of charge separation. This observation aligns with findings by Takata *et al.* (2020),[16] who reported that anisotropic facet exposure promotes directional charge migration toward separate reduction and oxidation sites, thus minimizing recombination and enabling nearly unity quantum efficiency in Al-doped $SrTiO_3$. Furthermore, Li *et al.* (2023)[17] demonstrated that Al-doping not only suppresses formation of $Ti^{3+}$ recombination centers but also induces surface band bending, both of which enhance charge separation in facet-engineered structures.

Moreover, in our previous work on undoped cubic and truncated $SrTiO_3$, we demonstrated that facet engineering led to distinct reaction pathways of acetone photooxidation, resulting in markedly

different photocatalytic efficiencies and reaction rates.[18] Al doping introduces pronounced differences in carrier dynamics between the two morphologies. Specifically, the truncated Al-doped $SrTiO_3$ exhibits significantly longer lifetimes compared to the cubic morphology, suggesting that aluminum incorporation more effectively enhances photoinduced charge separation in the truncated nanostructures.

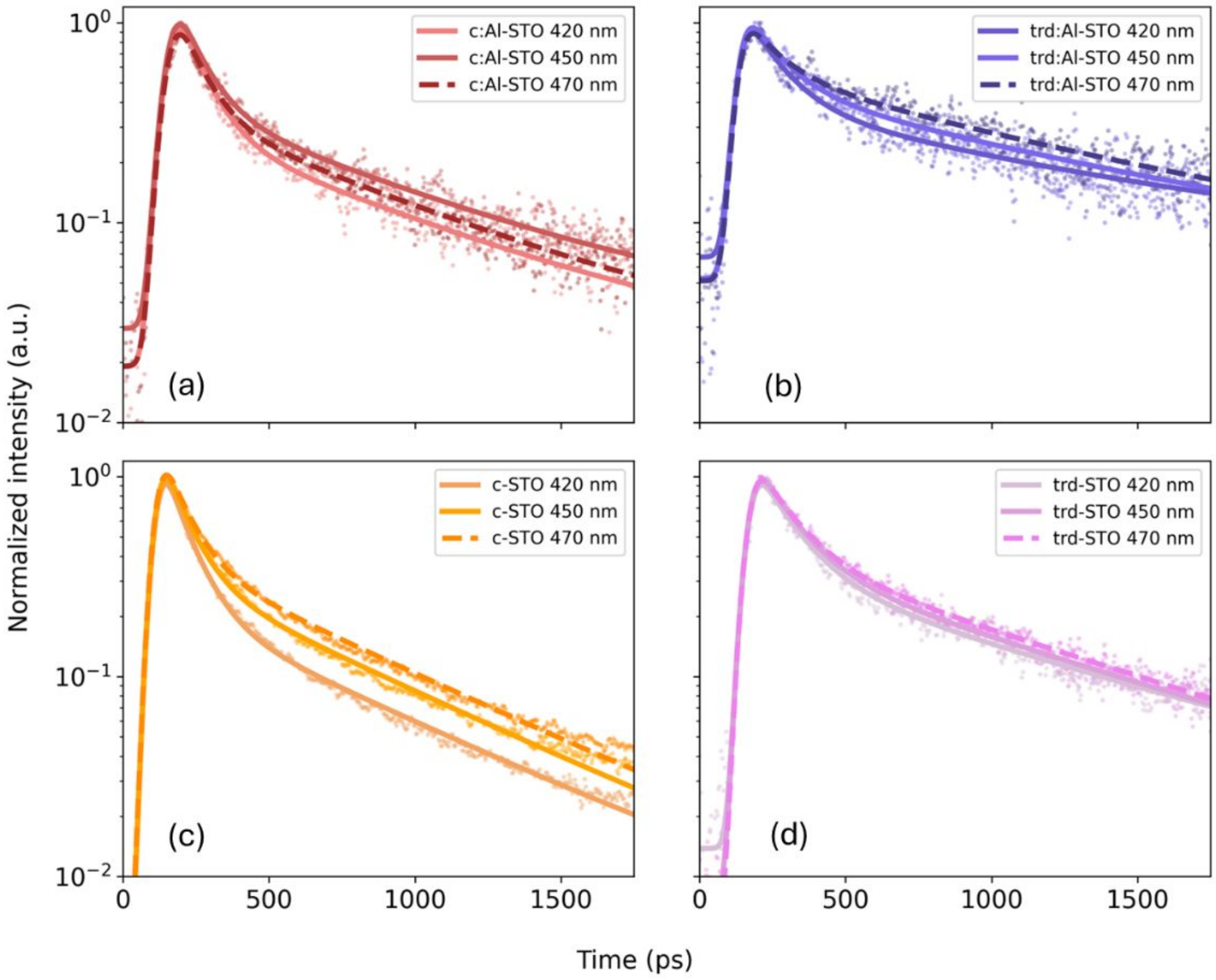


*Figure S7. Normalized PL decays at 420 nm, 450 nm, and 470 nm for (a) c-Al:STO, (b) trd-Al:STO, (c) c-STO and (d) trd-STO films following 267 nm excitation, including fits from global analysis using a sequential model with two components (solid and dashed lines).*

**References**


[1] P. Makuła, M. Pacia, W. Macyk, *Journal of Physical Chemistry Letters* **2018**, *9*, 6814.

[2] A. Mattsson, L. Österlund, *Journal of Physical Chemistry C* **2010**, *114*, 14121.

[3] H. Alalwan, A. Alminshid, *Spectrochim Acta A Mol Biomol Spectrosc* **2020**, *229*, 117990.

[4] D. M. Griffiths, C. H. Rochester, *Journal of the Chemical Society, Faraday Transactions 1: Physical Chemistry in Condensed Phases* **1978**, *74*, 403.

[5] T. Würger, W. Heckel, K. Sellschopp, S. Müller, A. Stierle, Y. Wang, H. Noei, G. Feldbauer, *Journal of Physical Chemistry C* **2018**, *122*, 19481.

[6] E. L. Jeffery, R. K. Mann, G. J. Hutchings, S. H. Taylor, D. J. Willock, *Catal Today* **2005**, *105*, 85.

[7] Y. Wang, B. Wen, A. Dahal, G. A. Kimmel, R. Rousseau, A. Selloni, N. G. Petrik, Z. Dohnálek, *Journal of Physical Chemistry C* **2020**, *124*, 20228.

[8] A. Mattsson, M. Leideborg, K. Larsson, G. Westing, L. Österlund, *Journal of Physical Chemistry B* **2006**, *110*, 1210.

[9] A. Mattsson, M. Leideborg, L. Persson, G. Westin, L. Österlund, *Journal of Physical Chemistry C* **2009**, *113*, 3810.

[10] F. P. Rotzinger, J. M. Kesselman-Truttmann, S. J. Hug, V. Shklover, M. Grätzel, *Journal of Physical Chemistry B* **2004**, *108*, 5004.

[11] C. N. Rusu, J. T. Yates, *Journal of Physical Chemistry B* **2000**, *104*, 12292.

[12] K. Kobl, L. Angelo, Y. Zimmermann, S. Sall, K. Parkhomenko, A. C. Roger, *Comptes Rendus Chimie* **2015**, *18*, 302.

[13] K. R. Phillips, S. C. Jensen, M. Baron, S. C. Li, C. M. Friend, *J Am Chem Soc* **2013**, *135*, 574.

[14] J. E. Rekoske, M. A. Barteau, *Langmuir* **1999**, *15*, 2061.

[15] Z. Topalian, B. I. Stefanov, C. G. Granqvist, L. Österlund, *J Catal* **2013**, *307*, 265.

[16] T. Takata, J. Jiang, Y. Sakata, M. Nakabayashi, N. Shibata, V. Nandal, K. Seki, T. Hisatomi, K. Domen, *Nature* **2020**, *581*, 411.

[17] R. Li, T. Takata, B. Zhang, C. Feng, Q. Wu, C. Cui, Z. Zhang, K. Domen, Y. Li, *Angewandte Chemie International Edition* **2023**, *62*, e202313537.

[18] N. T. Costa, D. M. Cunha, K. Zhu, A. Huijser, G. Katsoukis, K. Wenderich, J. Flapper, G. Mul, *Energy & Environmental Materials* **2025**, e12862.
